# A Density Functional Study on Ethylene Trimerization and Tetramerization Using Real Sasol Cr-PNP Catalysts

**DOI:** 10.3390/molecules28073101

**Published:** 2023-03-30

**Authors:** Minserk Cheong, Ajeet Singh

**Affiliations:** Department of Chemistry, Research Institute for Basic Sciences, Kyung Hee University, Seoul 02447, Republic of Korea

**Keywords:** DFT calculations, tri- and tetramerization of ethylene, real Sasol chromium complex

## Abstract

To gain molecular-level insight into the intricate features of the catalytic behavior of chromium–diphosphine complexes regarding ethylene tri- and tetramerizations, we performed density functional theory (DFT) calculations. The selective formation of 1-hexene and 1-octene by the tri- and tetramerizations of ethylene are generally accepted to follow the metallacycle mechanism. To explore the mechanism of ethylene tri- and tetramerizations, we used a real Sasol chromium complex with a nitrogen-bridged diphosphine ligand with *ortho*- and *para*-methoxyaryl substituents. We explore the trimerization mechanism for ethylene first and, later on for comparison, we extend the potential energy surfaces (PES) for the tetramerization of ethylene with both catalysts. The calculated results reveal that the formation of 1-hexene and 1-octene with the *ortho*-methoxyaryl and *para*-methoxyaryl Cr-PNP catalysts have nearly similar potential energy surfaces (PES). From the calculated results important insights are gained into the tri- and tetramerizations. The tetramerization of ethylene with the *para*-methoxyaryl Cr-PNP catalyst lowers the barrier height by ~2.6 kcal/mol compared to that of ethylene with the *ortho*-methoxyaryl Cr-PNP catalyst. The selectivity toward trimerization or tetramerization comes from whether the energy barrier for ethylene insertion to metallacycloheptane is higher than β-hydride transfer to make 1-hexene. The metallacycle mechanism with Cr (I)–Cr (III) intermediates is found to be the most favored, with the oxidative coupling of the two coordinated ethylenes to form chromacyclopentane being the rate-determining step.

## 1. Introduction

Linear α-olefins (LAOs) are valuable building blocks for a range of industrial and consumer products, including surfactants, comonomers, and synthetic lubricants [1,2,3,4,5,6,7]. At present, the bulk of LAOs is produced through metal-catalyzed ethylene oligomerization processes. The industrial use of linear α-olefins depends mainly on the chain length, with lower C_4_–C_8_ oligomers used as comonomers for ethylene polymerization. Several related processes are in use at present, with the most important being the production of high-density polyethylene (HDPE) and linear low-density polyethylene (LLDPE) [8].

Selective catalysts for the oligomerization of ethylene have been developed by Phillips [9]. Producing 1-hexene or 1-octene without any other by-products can have significant environmental benefits. These catalysts consist of a substituted pyrrole ligand, a chromium source, and an alkyl aluminum activator. These catalysts achieve the good selectivity of 1-hexene and have remained the state of the art for many years. Most research in the area has focused on minor modifications to these systems until very recently [10,11,12,13].

**Figure 1:**
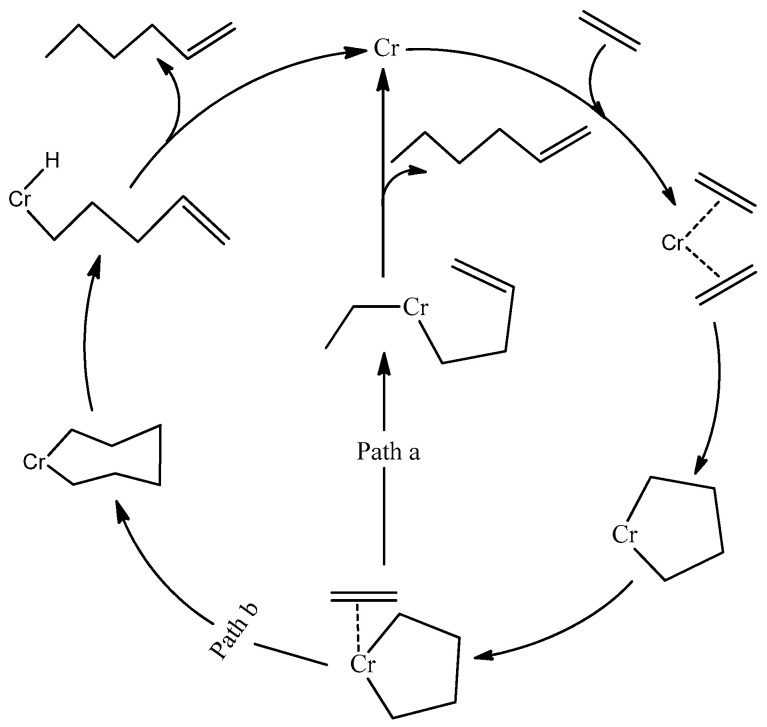
Proposed mechanism of ethylene trimerization.

Since the early discovery of a selective trimerization catalyst by Manyik et al. of the Union Carbide Corporation, several catalyst systems based on chromium and titanium have been developed [14]. The production of 1-hexene by these catalysts was explained by the same author, who proposed for the first time the involvement of metallacycles as key intermediates (Figure 1, Path a). This mechanism was expanded by Briggs [15] (Figure 1, Path b), who suggested the third ethylene molecule interacts with metallacyclopentane to yield metallacycloheptane instead of forming a dialkyl intermediate, as suggested by Manyik et al. Many experiments and density functional theory (DFT) studies have been performed to prove the metallacycle mechanism since then. Emrich et al. obtained the crystal structures of the intermediates and confirmed that the two metallacycles are involved in the mechanism. They also demonstrated that the metallacyclopentane is more stable than metallacycloheptane and that the latter decomposes with the liberation of 1-hexene [16]. Furthermore, Agapie et al. successfully distinguished the metallacycle mechanism for selective trimerization on a chromium-PNP catalyst from a Cossee-type mechanism [17,18] for non-selective ethylene oligomerization on a nickel-based catalyst through deuterium-labeling experiments [19,20]. Later, an NMR study by Arteaga-Müller et al. confirmed that metallacyclopentane and metallacycloheptane are important intermediates responsible for ethylene trimerization into 1-hexene [21].

Although extensive experimental and theoretical studies have been conducted, many important mechanistic details for trimerization remain unclear, although the metallacycle mechanism is widely accepted. The two proposed oxidation states of chromium are I/III or II/IV [22,23,24,25,26]. The variable oxidation states of organometallic complexes make computational calculations notoriously difficult, with SCF convergence problems and the computationally expensive nature of open shell calculations [27]. These phenomena are likely the reasons why theoretical studies based on Cr chemistry are less frequently studied than those based on other transition metals. Recent calculations emphasize the importance of spin state change during the reaction of transition metal compounds [28,29,30,31]. DFT studies by Budzelarr for a chromium indolate-AlR_2_Cl catalyst and Britovsek et al. and Gong et al. for chromium-PNP catalysts suggest that chromium (I/III) has a reasonable possibility for ethylene trimerization compared to chromium (II/IV) [32,33,34]. Not only the DFT study but also many other experimental studies on the Cr-PNP system have been considered the active species of Cr to be in the oxidation state I/III during the catalytic cycle [20,35,36]. In this study, we start calculations with chromium (I) and perform the spin state change of chromium from I to III during the course of the reaction.

**Figure 2:**
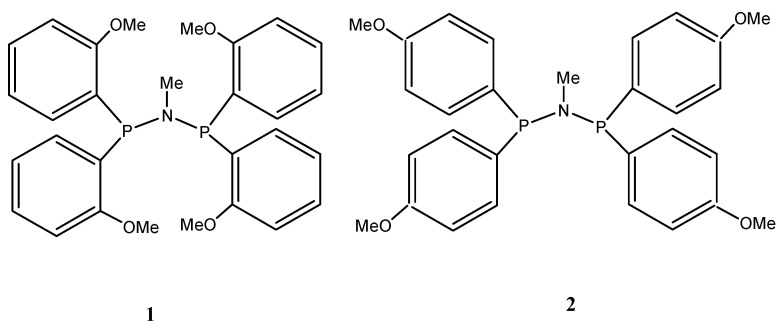
*Ortho*- and *para*-methoxyaryl PNP ligands.

A plethora of catalyst systems, mostly based on chromium [14,15,16,37,38,39,40,41,42,43,44,45,46,47,48], although others including titanium [49,50,51], nickel [52], and tantalum [53] are also known, exhibit a surprisingly high selectivity for the trimerization of ethylene to form 1-hexene. The most prominent among these is the chromium PNP catalyst system discovered by Wass et al., which comprises Ar_2_PN (Me) PAr_2_ (Ar = *ortho*-methoxy-substituted aryl group). Upon activation with methyl aluminoxane (MAO), the catalyst yielded highly selective 89.9 wt% 1-hexene with unprecedented productivity [40]. This figure of merit is about two orders of magnitude greater than previous systems under similar conditions. Furthermore, the experimental approach by Overett et al. to elucidate the role of methoxy substitution at the aromatic ring of the PNP ligand revealed that *ortho*-methoxy substitution plays a vital role in the catalytic performance for the selective trimerization of ethylene [54]. Changing the methoxy substitution from *ortho* to *meta* led to a drastic swing in chain-length distribution from predominantly 1-hexene to a significantly greater amount of 1-octene. This swing towards ethylene tetramerization was even more pronounced when the methoxy group was further moved to the *para* position [40]. The experimental mechanistic study provided evidence for metallacyclic mechanism as well as insights into the nature of the active site for such systems. However, there is a lack of common understanding regarding the high selectivity towards the trimerization of ethylene and the selectivity swing with substituent position with these (Cr-**1** and Cr-**2**, with ligands in Figure 2) catalysts.

Bis-ethylene *ortho*- and *para*-methoxyaryl Cr-PNP catalysts are our starting species, in which chromium has the oxidation state I (**1**R and **2**R). After Cr (I) is oxidized to Cr (III) (**1**SC1 and **2**SC1), a five-membered metallacycle is formed (**1**INT1 and **2**INT1). However, since no 1-butene is experimentally observed, the reaction is believed to continue to obtain **1**INT2 and **2**INT2, in which a third ethylene molecule is coordinated. After the ethylene insertion (**1**INT3 and **2**INT3), the reaction might proceed with a ring opening to finally produce 1-hexene. Alternatively, the reaction may proceed with the insertion of a new ethylene molecule to yield **1**INT4 and **2**INT4, which leads to 1-octene.

In the present theoretical study, we explore the tri- and tetramerization reactions of ethylene by applying density functional theory using *ortho*- and *para*-methoxyaryl Cr-PNP catalysts, to find a theoretical explanation for the observed experimental selectivity. To the best of our knowledge, no theoretical study with the real Sasol Cr-catalyzed mechanism for ethylene tri- and tetramerizations has been reported. In this DFT study, we use catalysts without any simplification to study the subtle steric effects more accurately, which are commonly lacking in previous theoretical studies [55], especially with Cr-PNP based catalysts.

## 2. Results and Discussion

### 2.1. Structure and Bonding Aspects of the Ortho-Methoxyaryl Cr-PNP Catalyst

**Figure 3:**
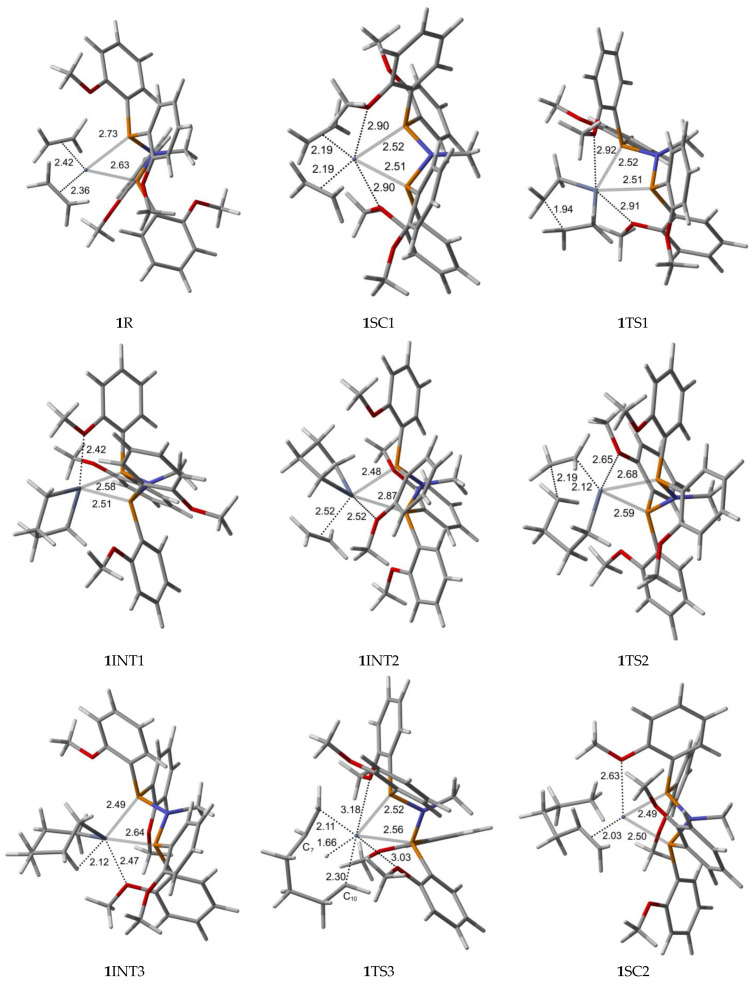

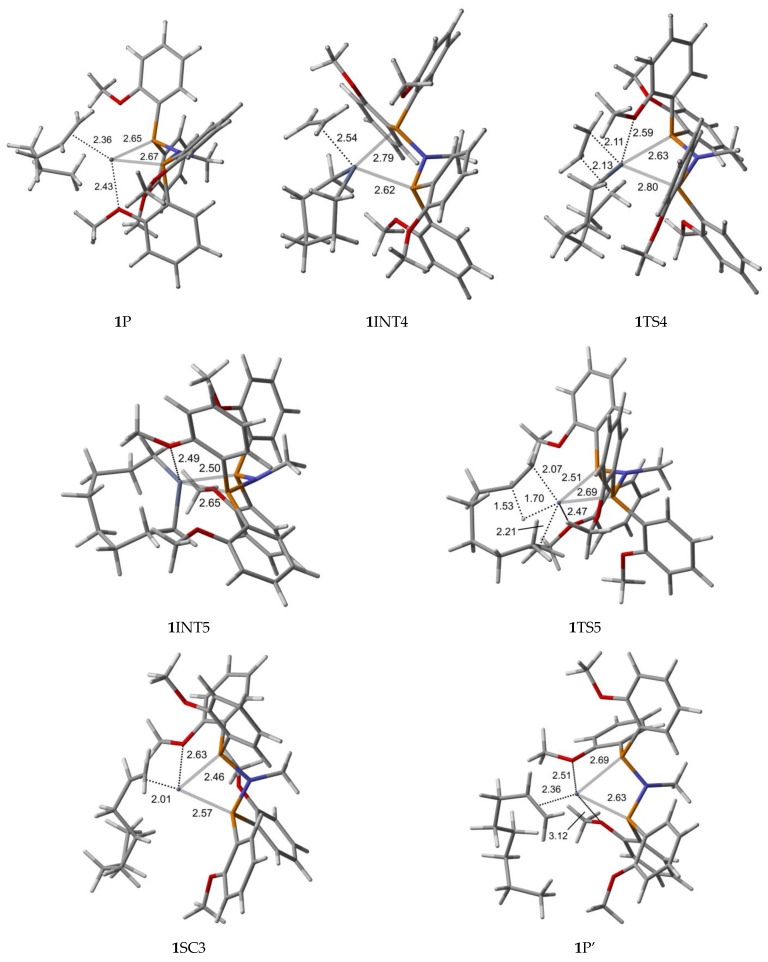
B3LYP/6-31G*/LanL2DZ optimized geometries (Supplementary Material) with important distances (Å) for ethylene trimerization and tetramerization using the *ortho*-methoxyaryl Cr-PNP catalyst. (Gray: carbon; red: oxygen; white: hydrogen; orange: phosphorus; blue: nitrogen; dark gray: Cr).

The optimized geometries for the ortho-methoxyaryl Cr-PNP catalyst participating in the ethylene trimerization and tetramerization mechanisms are presented in Figure 3. The Cr (I) sextet species **1**R is considered the first active complex in the tri- and tetramerization cycle for the ortho-methoxyaryl Cr-PNP catalyst. The optimized structure **1**R has a tetrahedral geometry with Cr-P distances of 2.631 and 2.733 Å. Initially, both ethylene molecules are weakly coordinated to Cr (I) via π-bonding interactions. The relatively weak nature of the π-bonding is reflected in the relatively shorter C-C bonding distance of 1.353 and 1.357 Å, which is 0.022–0.026 Å longer than the calculated C=C distance in free ethylene.

Furthermore, the Cr-C distances for both ethylene fragments are 2.363 and 2.418 Å, which indicates that both ethylenes are coordinated with nearly equal strength. During the catalytic cycle, it is known that the spin state of Cr changes [31,56], implying that at least one other stable structure exists between the reactants and the transition state. Therefore, we located the possible spin crossover quartet **1**SC1 complex between the reactant and first transition state **1**TS1. The calculated structure of complex **1**SC1 has an octahedral geometry with two of the four methoxy groups approaching from the apical positions with Cr-O distances of 2.897 Å and 2.904 Å and the two ethylene fragments are more tightly coordinated to Cr (I) with distances of 2.191 Å compared to **1**R. Two ethylene molecules moved to the same plane as the PNP ligand, making room for these two methoxy groups to coordinate with the chromium metal. This hemilabile behavior of the methoxy groups upon the coordination to Cr is vital in the catalytic activity and selectivity. The formation of the five-membered metallacyclic Cr (III) species **1**INT1 is afforded by the oxidative addition of the two ethylene fragments via the transition structure **1**TS1. In octahedral **1**TS1 with the apical Cr-O distances of 2.907 Å and 2.917 Å, the coupling C-C distance of the ethylenes decreases from 2.614 Å in **1**SC1 to 1.942 Å. The five-membered metallacycle product **1**INT1 has a C-C coupling distance of 1.536 Å, which agrees with the expected distance for the C-C single bond. Because of the position of the metallacyclic ring, the structure is trigonal bipyramidal. This coordination behavior of the ortho-methoxy group to Cr (III) has a profound steric effect and an electronic effect throughout the catalytic cycle. In order for ethylene trimerization to proceed, the incorporation of a third ethylene molecule is necessary. The interaction of ethylene with the trigonal bipyramidal five-membered metallacycle **1**INT1 affords octahedral **1**INT2, in which the ethylene fragment is weakly coordinated via a long-range π-interaction. This is evident from the elongated Cr-C distances of 2.522 Å for Cr-ethylene in **1**INT2, as well as the relatively short C=C distance of 1.345 Å for the coordinated ethylene fragment (Figure 3). Metallacycle growth from **1**INT2 to **1**INT3 via **1**TS2 yields the seven-membered metallacycle with *β*-agostic hydrogen. In order for the liberation of 1-hexene to proceed from the seven-membered metallacycles, **1**INT3, the reductive migration of this *β*-agostic hydrogen needs to take place. The Cr-mediated hydrogen migration from C7 in **1**TS3 to C10 yields 1-hexene, coordinated to the resulting quartet **1**SC2 with a Cr (I) oxidation state. In **1**TS3, the methoxy group, which previously coordinated to Cr, moves away from Cr, possibly because of the sterically crowded environment of the transition state. However, the other methoxy group moves in to compensate for the electronic requirement of the catalyst. Then, spin crossover from seesaw quartet **1**SC2 provides the more stable tetrahedral sextet **1**P, both with coordinated methoxy groups.

To investigate the possibility of tetramerization with this catalyst, we extended the PES for the next insertion of ethylene. The coordination of the fourth ethylene to **1**INT3 forms intermediate **1**INT4. In **1**INT4, the distance of C1 and C6 with Cr is 2.043 and 2.028 Å, respectively, while the newly coordinated ethylene is 2.536 Å away, indicating a weak Cr–ethylene interaction. The most important change in the coordination sphere is the absence of the methoxy group. The seven-membered metallacycle and ethylene are sterically crowded enough to move the methoxy groups out of the coordination sphere. The formation of the nine-membered metallacycle Cr(III) species **1**INT5 is achieved via the transition structure **1**TS4. In **1**TS4, the coupling distance of C-C decreases from 3.160 Å to 2.127 Å. Additionally, the coordination of the methoxy group occurs since the ethylene moiety moves closer to metallacycle to create space. The nine-membered metallacycle product **1**INT5 has a C-C coupling distance of 1.544 Å, which is in agreement with the expected distance for the C-C single bonds. Then, the migration of the *β*-agostic hydrogen yields seesaw **1**SC3 via **1**TS5, and the spin crossover produces the more stable trigonal bipyramidal sextet **1**P’.

### 2.2. Structure and Bonding Aspects of the Para-Methoxyaryl Cr-PNP Catalyst

**Figure 4:**
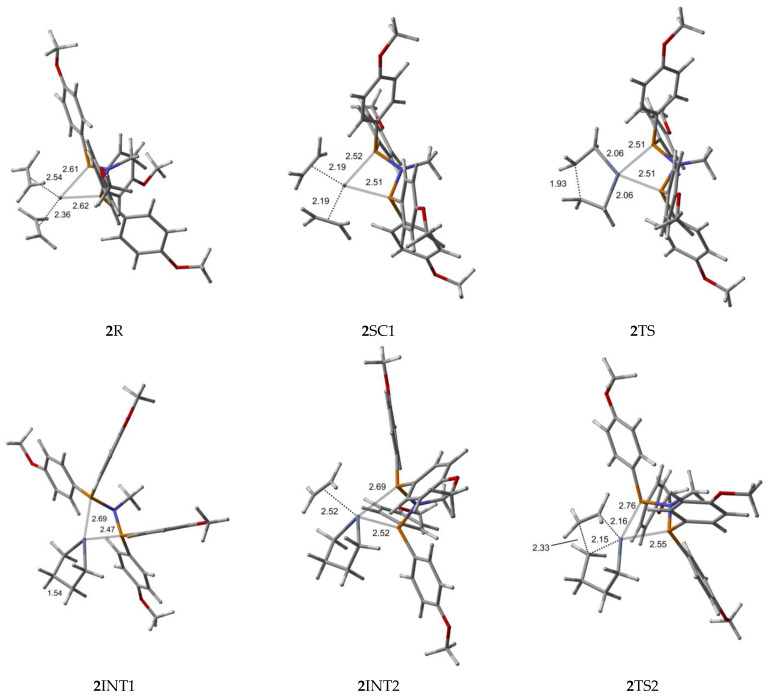

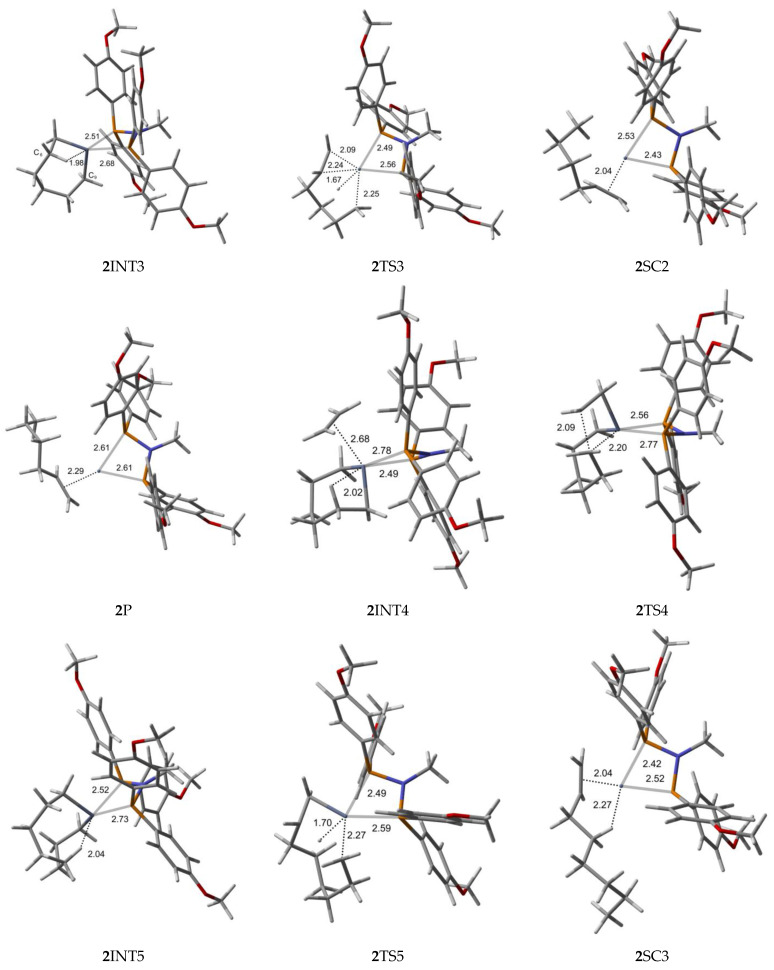

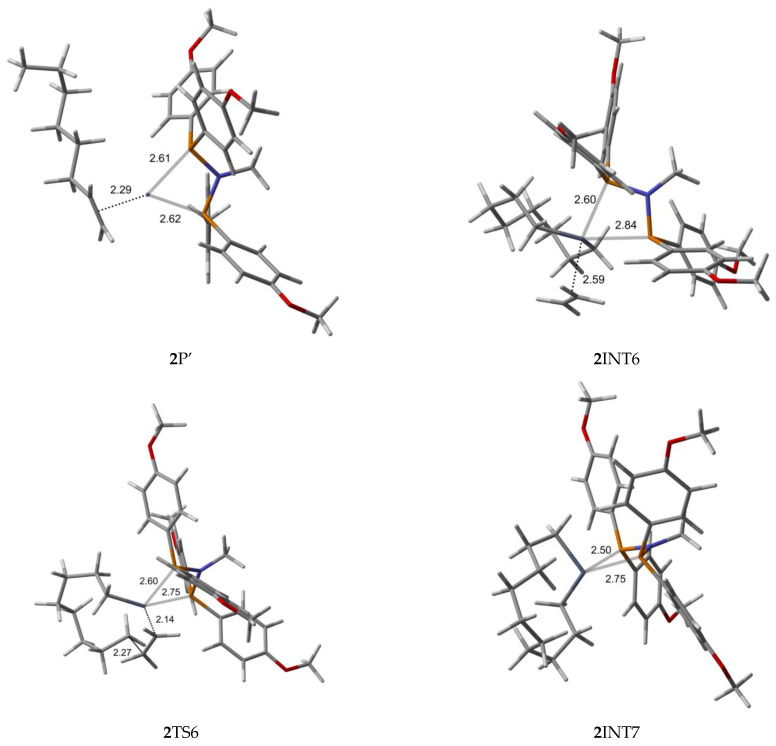
B3LYP/6-31G*/LanL2DZ optimized geometries (Supplementary Material) with important distances (Å) for ethylene trimerization and tetramerization using the *para*-methoxyaryl Cr-PNP catalyst. (Gray: carbon; red: oxygen; white: hydrogen; orange: phosphorus; blue: nitrogen; dark gray: Cr).

To explore the catalytic behavior of the para-methoxyaryl Cr-PNP catalyst, we performed calculations and located the stationary structures along the PES. All the optimized structures for trimerization and tetramerization are presented in Figure 4. Compared to the ortho-methoxyaryl Cr-PNP catalyst, there is no coordination of the methoxy group to chromium in the para-methoxyaryl Cr-PNP catalyst. This leads to a significant difference in the structures and energetics of the system.

The Cr (I) sextet species **2**R is considered the first active complex in the tri- and tetramerization cycle for the para-methoxyaryl Cr-PNP catalyst. The optimized structure **2**R has a tetrahedral geometry with Cr-P distances of 2.613 and 2.622 Å. Initially, both ethylene molecules are weakly coordinated to Cr(I) via π-bonding interactions. The relatively weaker nature of the π-bonding is reflected in the relatively shorter C-C bonding distances of 1.348 and 1.359 Å, which are 0.017–0.028 Å longer than the calculated C=C distance in free ethylene. Furthermore, the Cr-C distances for both ethylene fragments are 2.355 and 2.542 Å, indicating that both ethylene fragments are coordinated with nearly equal strength. As in the ortho-methoxyaryl Cr-PNP catalyst, we located the possible spin crossover quartet **2**SC1 complex between the reactant and first transition state **2**TS1. The calculated structure of complex **2**SC1 has a square-planar geometry, and the two ethylene fragments are more tightly coordinated to Cr (I) with distances of 2.19 Å compared to **2**R. The formation of the five-membered metallacycle Cr (III) species **2**INT1 is afforded by the oxidative addition of the two ethylene fragments via the transition structure **2**TS1. In square-planar **2**TS1, the C-C coupling distance of the ethylenes decreases from 2.611 Å in **2**SC1 to 1.930 Å. The five-membered metallacycle product **2**INT1 has C-C coupling distances of 1.539 Å, which is in agreement with the expected distance for C-C single bonds. In order for ethylene trimerization to proceed, the incorporation of a third ethylene molecule is necessary. The interaction of ethylene with the square-planar five-membered metallacycle **2**INT1 affords square-pyramidal **2**INT2, in which the ethylene fragment is weakly coordinated via a long-range π-interaction. This is evident from the elongated Cr-C distances of 2.521 Å for Cr- ethylene in **2**INT2, as well as the relatively short C=C distance of 1.347 Å for the coordinated ethylene fragment (Figure 4). Metallacycle growth from **2**INT2 via **2**TS2 to **2**INT3 yields the seven-membered metallacycle. To proceed with the liberation of 1-hexene from the seven-membered metallacycle (**2**INT3), reductive hydrogen migration from C6 to C9 is required. The migration of the *β*-agostic hydrogen of C6 in **2**INT3 to C9 leads to the formation of an agostic intermediate structure via the transition structure **2**TS3. The Cr-mediated hydrogen migration from C6 in **2**TS3 to C9 yields 1-hexene, coordinated to the resulting T-shaped quartet **2**SC2 with Cr (I) oxidation state. Then, spin crossover occurs and yields a more stable trigonal planar sextet **2**P.

To explore the possibility of tetramerization with this catalyst, we extended the PES for the next insertion of ethylene. The coordination of the fourth ethylene to **2**INT3 forms an intermediate **2**INT4. In **2**INT4, the distance between Cr and the newly coordinated ethylene is 2.678 Å, indicating a weak Cr–ethylene interaction. The formation of the nine-membered metallacycle Cr (III) species **2**INT5 is achieved via the transition structure **2**TS4. In **2**TS4, the C-C coupling distance decreases from 3.384 Å to 2.086 Å. The nine-membered metallacycle products **2**INT5 have C-C coupling distances of 1.545 Å, which agrees with the expected distance for a C-C single bond. Then, the migration of the *β*-agostic hydrogen yields **2**SC3 via **2**TS5, and the spin crossover produces a more stable sextet **2**P’.

Furthermore, we explored the possibility of pentamerization for the reasons explained in the subsequent section. The coordination of the fourth ethylene to **2**INT5 forms an intermediate **2**INT6. The formation of the eleven-membered metallacycle Cr(III) species **2**INT7 is achieved via the transition structure **2**TS6.

### 2.3. Energetic Aspects of the Cr-PNP Catalyst

**Figure 5:**
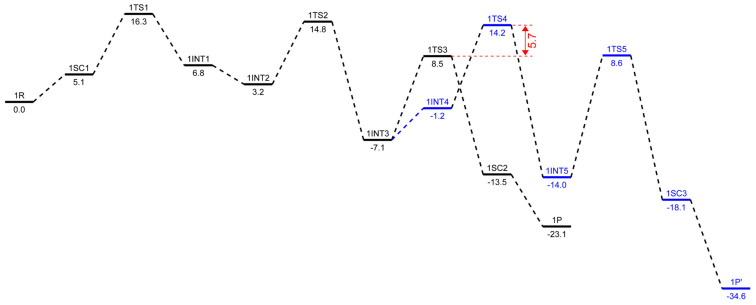
Corrected free energy profiles (in kcal/mol) at the level of B3LYP/6-311++G(d,p) with GD3BJ for ethylene trimerization in black and tetramerization in blue, using the *ortho*-methoxyaryl Cr-PNP catalyst. The SMD polarizable continuum solvent model was used for solvent effect correction.

**Figure 6:**
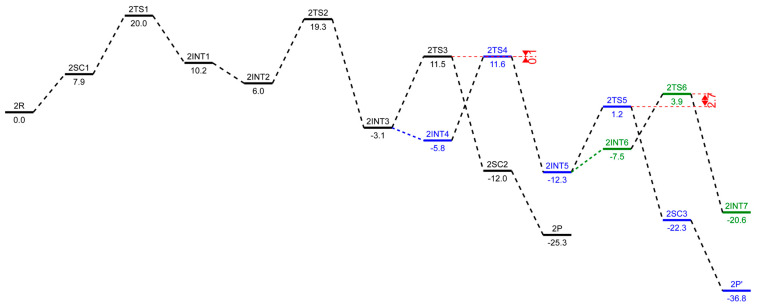
Corrected free energy profiles (in kcal/mol) at the level of B3LYP/6-311++G(d,p) with GD3BJ for ethylene trimerization in black, tetramerization in blue, and petamerization in green using the *para*-methoxyaryl Cr-PNP catalyst. The SMD polarizable continuum solvent model was used for solvent effect correction.

The structures **1**R and **2**R were chosen as a reference structure, and the energies of the following intermediates, transition states, and products are related to **1**R and **2**R, corrected with the right number of ethylene molecules. In Figure 5 and Figure 6, free energy profiles (in kcal/mol) are plotted for the stationary points along the reaction pathway. The energies for the optimized structures were corrected by triple-zeta single-point calculations at the 6-311++G(d,p) level of theory with Grimme’s D3 empirical dispersion corrections, including Becke–Johnson damping. Additionally, corrections for reactions in the liquid phase were conducted using the SMD polarizable continuum solvent model augmented with parameters for toluene. The conversions of the *bis*-ethylene complexes **1**R and **2**R to the corresponding metallacyclopentanes, **1**INT1 and **2**INT1, are endothermic reactions by 6.8 kcal/mol and 10.2 kcal/mol, respectively. The formal oxidative coupling of two ethylene molecules occur via the transition states **1**TS1 and **2**TS1 with activation energy barriers of 16.3 kcal/mol and 20.0 kcal/mol, respectively. The extra stabilization of 3.4–3.7 kcal/mol of the *ortho*-methoxyaryl Cr-PNP catalyst compared to that of the *para*-methoxyaryl Cr-PNP catalyst is due to the coordination of the *ortho*-methoxy group to the chromium metal ion.

This effect persists until the catalytic cycle reaches **1**INT4 and **2**INT4, at which point ethylene coordinates to metallacycloheptane, making the coordination sphere crowded enough to prevent the coordination of the *ortho*-methoxy group. This raises the energy of **1**INT4 compared to **2**INT4. The experimental evidence indicates that, with the Cr-PNP catalytic system, the rate-determining step is the oxidative coupling of the first two ethylene molecules to form the metallacyclopentane intermediate [57]. Our calculated result for both the *ortho*- and *para*-methoxyaryl Cr-PNP catalysts reaches a similar conclusion, with the activation energies of 16.3 and 20.0 kcal/mol, respectively, which are in good agreement with the experimental results.

There is a fair chance that the chromacyclopentane undergoes a ring-opening reaction, but no experimental evidence has been found for the formation of 1-butene with these catalysts, so we did not attempt to locate the transition state for ring opening with a five-membered metallacycle ring. The third ethylene molecule was added to the intermediate **1**INT1 or **2**INT1 to form **1**INT2 or **2**INT2, and both processes were downhill by 3.6 and 4.2 kcal/mol, respectively. The subsequent conversion of **1**INT2 or **2**INT2 to the intermediate **1**INT3 or **2**INT3 occurs via the insertion of ethylene into the Cr-C bond through the transition state **1**TS2 or **2**TS2, respectively. However, both intermediates stabilize at nearly the same energetic values and are also exothermic in nature with −10.3 and −9.1 kcal/mol, respectively. The activation barriers for both **1**INT2-**1**INT3 and **2**INT2-**2**INT3 are 14.8 and 19.3 kcal/mol, respectively. The conversion of the intermediate **1**INT3 or **2**INT3 to produce 1-hexene occurs via an agnostic-assisted hydride transfer [58,59]. The transition states (**1**TS3 and **2**TS3) associated with these conversions have activation energy barriers of 8.5 and 11.5 kcal/mol, respectively. The products **1**SC2 and **2**SC2 are the most stable intermediates in their respective catalytic cycle, with relative energies of −13.5 and −12.0 kcal/mol, respectively. We also located the spin crossover complexes with Cr(I) (**1**P and **2**P), which are very low in energy, with −23.1 and −25.3 kcal/mol, respectively, compared to the Cr (III) oxidation state intermediates (**1**SC2 and **2**SC2).

The calculated results of the trimerization with the *ortho*- and *para*-methoxyaryl Cr-PNP catalysts indicate that both tend to form 1-hexene, which was also observed experimentally, even though with the *para*-methoxyaryl Cr-PNP catalyst, the percentage of trimerization product is low. To address the question of why there is a selectivity change or shift from trimerization to tetramerization with the shifting of the –OMe substituent on the ligand backbone, we extended the PES and located the stationary points with the fourth molecule of ethylene. The subsequent conversion of **1**INT3 or **2**INT3 to the intermediate **1**INT4 or **2**INT4 occurs via the coordination of another ethylene molecule. There is a great difference in energy between the *ortho*-methoxyaryl Cr-PNP catalyst and the *para*-methoxyaryl Cr-PNP catalyst. In the case of the *para*-methoxyaryl Cr-PNP catalyst, it is an exothermic reaction by 2.7 kcal/mol, as in the conversion of **2**INT1 to **2**INT2. However, in the case of the *ortho*-methoxyaryl Cr-PNP catalyst, the reaction becomes endothermic by 5.9 kcal/mol. The reason for the considerable difference comes from the absence of the coordination of the *ortho*-methoxy group in moving from **1**INT3 to **1**INT4 because of steric crowdedness in the coordination sphere. This steric effect also affects the insertion of ethylene into the Cr-C bond via the transition state **1**TS4 (Figure 5). The activation barriers of both 1INT4-1INT5 and 2INT4-2INT5 are 14.2 and 11.6 kcal/mol, respectively. This shows that the *ortho*-methoxyaryl Cr-PNP catalyst suffers more steric hindrance than the *para*-methoxyaryl Cr-PNP catalyst, as expected. Energy barriers for the metallacycle expansion of ring from seven- to nine-membered (chromium-cyclononane) are very high. Such an exceptionally high energy can be explained by means of steric effects and the instability of the nine-membered ring. The calculated results indicate that the formation of 1-hexene is kinetically preferred with both catalysts, while the formation of 1-octene with these catalysts seems to be unfavored, even though with the *para*-methoxyaryl Cr-PNP catalyst, the energy barrier for tetramerization is lowered by ~2.7 kcal/mol than that of *ortho*-methoxyaryl Cr-PNP catalyst, making it almost the same as the energy barrier for trimerization. Therefore, the key to selectivity toward trimerization or tetramerization depends on the difference in transition state energy between ring expansion and β-hydride transfer. In the case of the *ortho*-methoxyaryl Cr-PNP catalyst, the β-hydride transfer is lower in energy than ring expansion by 5.7 kcal/mol (shown in red in Figure 5). This energy difference is large enough to prevent ring expansion, so experimentally, 82~91% selectivity toward 1-hexene is observed [20]. In the case of the *para*-methoxyaryl Cr-PNP catalyst, the energy difference is almost zero (shown in red in Figure 6). So, experimentally, only 38–50% selectivity toward 1-octene is observed [20], since both trimerization and tetramerization can occur. These calculation results surprisingly coincide with the experimental results very well. This can only happen with extensive conformational search and accurate energy calculation with dispersion and damping. The conversion of intermediate **1**INT5 or **2**INT5 to produce 1-octene occurs via agnostic-assisted hydride transfer. Both transition states (**1**TS5 and **2**TS5) associated with these conversions have activation energy barriers of 8.6 and 1.2 kcal/mol, respectively. The products **1**SC3 and **2**SC3 are the most stable intermediates in their respective catalytic cycles, with relative energies of −18.1 and −22.3 kcal/mol, respectively. We also located the spin crossover complexes with Cr (I) (**1**P’ and **2**P’), which are very low in energy, with −34.6 and −36.8 kcal/mol, respectively.

In case of the *para*-methoxyaryl Cr-PNP catalyst, both trimerization and tetramerization can occur. So, we have to investigate whether ring expansion can occur further. The conversion of **2**INT5 to the intermediate **2**INT6 occurs via the coordination of another ethylene. The reaction is endothermic by 4.8 kcal/mol, which is different from the same ethylene coordination from **2**INT3 to **2**INT4. The reason for the difference comes from the steric crowdedness in the coordination sphere with the larger metallacycle. This steric effect also affects the insertion of ethylene into the Cr-C bond via the transition state **2**TS6 (Figure 6) with the activation barriers of 3.9 kcal/mol. The energy barrier for the metallacycle ring expansion from nine to eleven is higher than that of the β-hydride transfer to produce 1-octene by 2.7 kcal/mol (shown in red in Figure 6). Therefore, we can expect that the further expansion of metallacycle to an 11-membered ring is unfavorable, as shown in the experimental results.

The experimental results show that changing from *ortho*-methoxy to *para*-methoxy in the catalyst backbone causes a shift in product distribution from 1-hexene only to a mixture of 1-octene and 1-hexene. In this study, we attempted to explain this mechanism using DFT calculations and found out that, with the *ortho*-methoxyaryl PNP ligand, the methoxy group is in close proximity to the chromium metal for coordination and plays a major role in the catalytic cycle, resulting in the production of only 1-hexene. Conversely, with the *para*-methoxyaryl PNP ligand, this coordination does not occur, and the product distribution is determined solely by steric hindrance from the phenyl group, resulting in an almost 50:50 ratio of 1-hexene to 1-octene.

## 3. Computational Methods

Geometries were fully optimized using the non-local B3LYP method, which is a combination of Becke’s three-parameter hybrid exchange functional and the correlation functional of Lee, Yang, and Parr [60,61,62], without any symmetry constraints. The LANL2DZ (Los Alamos National Laboratory second double-zeta) basis set was used for Cr atoms, while the 6-31G* basis set was used for C, H, N, O and P atoms [63,64], with the GAUSSIAN 16 program package [65]. Harmonic force constants were computed for the optimized geometries to characterize the stationary points as minima. The number of imaginary frequencies (Imag) at the same level of theory, i.e., energy minimum structures without imaginary frequencies (NImag = 0) and transition states with only one imaginary frequency (NImag = 1), confirmed whether the optimized structures are transition states or true minima on the potential energy surfaces. The magnitude of the imaginary frequency and the corresponding eigenvectors were analyzed for all transition states to verify the involvement of the required atoms.

Exhaustive conformational searching was performed for all ground-state and transition state structures, but only the lowest-energy structures are reported. The energies of reactants, transition states, and products were corrected by triple-zeta single-point calculation at the 6-311++G(d,p) level of theory. Dispersion corrections were included using the D3 empirical correction from Grimme [66], which includes Becke–Johnson damping [67]. Additionally, corrections for reactions in the liquid phase were made using the SMD polarizable continuum solvent model [68], augmented with parameters for toluene.

## 4. Conclusions

The selective nature of Cr-PNP catalysts for the tri- and tetramerizations of ethylene is of immense interest to experimental and theoretical chemists. However, theoretical studies on Cr-PNP catalysts are scarce in the literature. We carried out a DFT study using the real Sasol catalyst following the metallacycle mechanism, which has already been established by many experimental and theoretical studies. In this DFT study, we considered the oxidation state of Cr as I/III. The Gibbs free energy profile at 298.15K for the full potential energy surfaces of the *ortho*- and *para*-methoxyaryl Cr-PNP catalysts indicates that the trimerization of ethylene is kinetically feasible in both cases. However, the ligand backbone seems to play a vital role towards the selectivity. The propensity for trimerization is caused by the hemilabile *ortho*-methoxy group with steric hindrance. The swing from the tri- to tetramerization of ethylene with the *para*-methoxyaryl Cr-PNP ligand seems to be caused by moving the *ortho*-methoxy group out of the coordination sphere.

## Data Availability

Data are available within the article and the Supplementary Materials.

## Supplementary Materials

The following supporting information can be downloaded at: https://www.mdpi.com/article/10.3390/molecules28073101/s1. Optimized coordinates of the DFT calculations.
