# Supplementary material for: A Density Functional Study on Ethylene Trimerization and Tetramerization Using Real Sasol Cr-PNP Catalysts
Source: Molecules. 2023 Mar 30;28(7):3101. doi: 10.3390/molecules28073101 (PMC10095999; doi:10.3390/molecules28073101)
Supplement: Supplementary file 1 [file molecules-28-03101-s001.zip › molecules-2321228-supplementary.pdf]

Supplementary Material

A Density Functional Study on Ethylene Trimerization and Tetramerization Using Real Sasol Cr-PNP Catalyst

Minserk Cheong * and Ajeet Singh

Department of Chemistry and Research Institute for Basic Sciences, Kyung Hee University, Seoul 02447, Republic of Korea

Cartesian Coordinates (Å) of B3LYP/6-31G*/LanL2DZ Optimized Geometries in Figures 3 and 4

1R

```
N -0.04670600 0.20396100 -1.00746100
P 1.28325600 -0.05068200 0.07383300
P -1.56445600 0.11172700 -0.14939400
Cr -0.25086200 -0.59025600 2.14317900
C -0.45554600 1.25724100 3.75855200
C -1.29954600 0.28310000 4.18214900
C -0.21906800 -3.05555200 1.75118900
C -0.70394400 -2.91853400 3.00692500
C 0.06011600 0.85032000 -2.33094100
C 2.24931100 -1.46621900 -0.59125000
C 2.42094700 1.39257500 0.00583500
C -2.52696600 1.57081300 -0.72651400
C -2.46598500 -1.30897500 -0.90221700
C 3.27937700 -1.98473600 0.22484600
C 4.01166100 -3.10645600 -0.17761500
C 3.71479600 -3.72344000 -1.39533300
C 1.96786900 -2.11236000 -1.79830000
C 2.69278900 -3.23394800 -2.20661500
O 3.47711400 -1.32470600 1.40654200
C 2.46176200 2.20052400 1.15115800
C 3.32204900 3.29318700 1.25648100
C 4.17046800 3.58767900 0.19043700
C 3.29785400 1.70331800 -1.06238900
C 4.16364700 2.80111100 -0.96190200
O 3.24591200 0.89601800 -2.15453500
C -2.18116700 2.85907500 -0.25635800
C -2.92546900 3.97796100 -0.64627900
C -4.01536400 3.82430200 -1.50534000
C -3.62333400 1.45168600 -1.59102600
C -4.36831900 2.56436900 -1.98252400
O -1.09236200 2.93304800 0.57023700
C -1.96929800 -2.07634100 -1.96105100
C -2.65185700 -3.20529800 -2.42208400
C -3.84845400 -3.57921800 -1.81418600
C -3.67407800 -1.71423600 -0.28828600
C -4.36457000 -2.84052800 -0.74560500
O -4.08494000 -0.93290700 0.75532100
C 4.56257600 -1.71795300 2.24091200
C 4.19552600 1.07796800 -3.20228900
C -0.61989200 4.22665500 0.94381400
C -5.35276300 -1.19250800 1.35161100
H 0.53466600 1.37286100 4.19404600
H -1.01865500 -0.40824600 4.97289600
H -2.32975900 0.23858600 3.83475400
H -0.88010400 -3.11444600 0.88968900
H -1.77315000 -2.86873900 3.20033700
H -0.05172300 -2.96169800 3.87664500
H -0.77899100 2.01283800 3.04719800
H 0.84115700 -3.20572600 1.56309800
H 4.80611800 -3.50250300 0.44429000
H 4.28763900 -4.59384000 -1.70205400
H 1.17758300 -1.72868900 -2.43330400
H 2.46185500 -3.71667300 -3.15131600
H 1.80907000 1.94837000 1.98249700
H 3.33847900 3.89650000 2.15910700
H 4.85098900 4.43241200 0.24979800
H 4.83418500 3.04753100 -1.77663200
H -2.66759400 4.96682600 -0.28714300
H -4.58530400 4.70125200 -1.79882000
H -3.89435300 0.47191000 -1.96952700
H -5.21144800 2.44519400 -2.65580200
H -1.03352500 -1.78953300 -2.42703600
H -2.25188000 -3.78224100 -3.25013800
```

H -4.39032000 -4.45284200 -2.16472600
H -5.29417800 -3.14748600 -0.28049700
H 1.00529000 0.57688400 -2.79947500
H -0.76552800 0.52738000 -2.97115900
H 0.01915900 1.94236100 -2.24294200
H 4.43485500 -2.74375700 2.60823300
H 5.51858100 -1.63615100 1.71069000
H 4.55534000 -1.02632600 3.08447800
H 5.22145900 1.00114300 -2.82435500
H 4.00839000 0.27009000 -3.91062500
H 4.05852700 2.04370600 -3.70336300
H 0.30316100 4.05887200 1.49842300
H -0.39949600 4.83606100 0.05994700
H -1.34577600 4.74959200 1.57818800
H -5.37558700 -2.17883800 1.83140200
H -5.49168400 -0.41710000 2.10618500
H -6.15839300 -1.12793900 0.61100300

1SC1

N -0.00326500 -0.02875300 -1.29708900
P 1.36349300 -0.07488800 -0.24349200
P -1.37010900 0.05917200 -0.24722000
Cr 0.00195900 -0.01068800 1.86510800
C 0.01269900 -0.07000700 -2.76683400
C 2.20378000 -1.64536300 -0.66302800
C 2.45876200 1.29585800 -0.78081600
C -2.19060300 1.64302200 -0.66183500
C -2.49393000 -1.28509700 -0.79281100
C 1.83942700 -2.80702500 0.05389300
C 2.40932900 -4.04234900 -0.27170700
C 3.33402800 -4.12470800 -1.31530500
C 3.13406800 -1.75869100 -1.70406100
C 3.69898500 -2.98956900 -2.03753800
O 0.91300600 -2.64634100 1.05602900
C 1.90696100 2.39724000 -1.45201700
C 2.68253900 3.50645700 -1.79024900
C 4.03247600 3.52767200 -1.44337900
C 3.81976400 1.35436000 -0.39707200
C 4.60257000 2.46204700 -0.74554700
O 4.28579600 0.29558300 0.32118000
C -1.80267400 2.80032700 0.04959100
C -2.35546100 4.04401000 -0.27388400
C -3.29009000 4.13826900 -1.30750500
C -3.13213500 1.76838400 -1.69084200
C -3.68188000 3.00661700 -2.02118200
O -0.87054900 2.62744400 1.04514100
C -1.98310000 -2.37426800 -1.51412800
C -2.78885500 -3.45944300 -1.86038100
C -4.12772800 -3.46780700 -1.47333800
C -3.84587000 -1.32867400 -0.37526100
C -4.65845400 -2.41199700 -0.73060100
O -4.27322800 -0.27810100 0.37824900
C 0.47629900 -3.80402600 1.77127300
C 5.65727500 0.26987400 0.71179200
C -0.40350300 3.78137000 1.74724800
C -5.63410200 -0.23509000 0.80316400
H 2.14360700 -4.93915600 0.27465800
H 3.76855600 -5.08983800 -1.55930900
H 3.42351000 -0.86742500 -2.25302600
H 4.41684500 -3.05944400 -2.84869300
H 0.85223200 2.38827300 -1.70582100
H 2.23601500 4.34060400 -2.32233700
H 4.65163700 4.38082400 -1.70512900
H 5.64910100 2.50378500 -0.46822600
H -2.06938300 4.93795300 0.26686800
H -3.71179900 5.10950300 -1.54977600
H -3.44291000 0.88077900 -2.23414600
H -4.40879600 3.08525400 -2.82343900
H -0.93802600 -2.37396700 -1.80464300
H -2.37365400 -4.28502600 -2.42983000
H -4.76947100 -4.30252800 -1.74007000
H -5.69768600 -2.44225700 -0.42573300
H 0.41098400 0.85502800 -3.19466000
H 0.62717500 -0.90800000 -3.11461300
H -1.00389300 -0.21439900 -3.13874600
H 0.00610500 -4.52791800 1.09666800
H 1.30874100 -4.27682100 2.30427600
H -0.26038400 -3.44767500 2.49205300
H 5.89607500 1.10670400 1.37865000
H 5.79377700 -0.67168800 1.24458300
H 6.31867200 0.29376600 -0.16200100
H 0.33141700 3.41546300 2.46498600
H 0.07646300 4.48915400 1.06254200
H -1.22115400 4.27599200 2.28325000
H -5.87298500 -1.08226900 1.45676400
H -5.73918400 0.69646100 1.36012500
H -6.31660600 -0.22624000 -0.05448900
C 2.00138300 0.62089300 2.67862300

H 2.73591900 -0.13124400 2.40829600
H 2.15003600 1.59897400 2.22586000
C 1.22087400 0.47538100 3.80858800
H 1.33929100 -0.38701200 4.45578400
H 0.74429800 1.33763800 4.26588200
C -1.20120400 -0.50796900 3.81628000
H -0.72084800 -1.37141000 4.26728800
H -1.31530500 0.35229200 4.46713200
C -1.98840500 -0.64930600 2.69066200
H -2.72385900 0.10392600 2.42672400
H -2.13896100 -1.62559200 2.23444400

1TS1

N 0.00571300 -0.00248800 -1.34559900
P 1.36538100 -0.09864100 -0.28717000
P -1.36294200 0.10292500 -0.29948500
Cr 0.00266000 0.02236600 1.81637500
C 0.02539700 -0.06681500 -2.81517400
C 2.15065000 -1.69946700 -0.69006400
C 2.51688600 1.23260100 -0.79411600
C -2.16828300 1.68955100 -0.72047200
C -2.50698500 -1.23578900 -0.80437100
C 1.66873700 -2.85113800 -0.02766800
C 2.19330200 -4.11061200 -0.33483300
C 3.19149600 -4.22576800 -1.30526000
C 3.15164200 -1.84544000 -1.65813000
C 3.67331800 -3.10036600 -1.97228300
O 0.67804300 -2.65325000 0.90340900
C 2.04458400 2.33455200 -1.52124500
C 2.87744000 3.41099900 -1.82933800
C 4.20157400 3.39776200 -1.39360500
C 3.84997700 1.25685400 -0.32066500
C 4.69015100 2.33107400 -0.63739800
O 4.22722500 0.19870300 0.44723900
C -1.68919100 2.85685900 -0.08381000
C -2.23078600 4.10580600 -0.40358700
C -3.24627200 4.19456500 -1.35886800
C -3.18945700 1.80892000 -1.67055000
C -3.72936600 3.05296200 -1.99663000
O -0.68384000 2.68326200 0.83661600
C -2.05324200 -2.31135500 -1.58054500
C -2.88748000 -3.38780100 -1.88613500
C -4.19434900 -3.39979600 -1.40184200
C -3.82450700 -1.28275700 -0.28967300
C -4.66588500 -2.35652600 -0.60290400
O -4.18557500 -0.24241500 0.50958300
C 0.07594400 -3.79755000 1.51333700
C 5.56231900 0.14128300 0.94498100
C -0.09346500 3.84237300 1.42952800
C -5.50455900 -0.20576000 1.05034000
H 1.83663700 -5.00011500 0.17027400
H 3.59083500 -5.20915900 -1.53615300
H 3.52779200 -0.96284400 -2.16696000
H 4.44696000 -3.19674400 -2.72749600
H 1.00810200 2.35475000 -1.84015400
H 2.49488900 4.24676900 -2.40682800
H 4.86384800 4.22525800 -1.63110800
H 5.71684900 2.34644300 -0.29147700
H -1.87464600 5.00729300 0.08022600
H -3.65907200 5.16992900 -1.59990100
H -3.56787800 0.91416500 -2.15602000
H -4.51827700 3.12854500 -2.73830300
H -1.03234400 -2.31090400 -1.94540800
H -2.51907100 -4.20359800 -2.50017900
H -4.85705500 -4.22775700 -1.63653600
H -5.68033000 -2.38919100 -0.22363600
H 0.51432500 -0.98709300 -3.15467200
H -0.99925700 -0.05918700 -3.19337000
H 0.55795800 0.78584500 -3.24616800
H -0.37282100 -4.45153700 0.75791700
H 0.80528700 -4.35818600 2.10858900
H -0.70554900 -3.40768000 2.16614200
H 5.77526400 0.98812400 1.60791900
H 5.62622000 -0.78923700 1.50973300
H 6.29019600 0.12173000 0.12554300
H 0.69702800 3.47071600 2.08208700
H 0.34253700 4.49354600 0.66425200
H -0.82686600 4.40033800 2.02232100
H -5.68986500 -1.06723700 1.70263400
H -5.55771900 0.71280700 1.63536600
H -6.25799400 -0.17608500 0.25468500
C 1.78779500 0.32577100 2.80385200
H 2.54205500 -0.39483900 2.49540600
H 2.12753800 1.35968600 2.71236300
C 0.97992100 -0.01181700 3.96904900
H 1.11095000 -1.03696200 4.31455900
H 1.02117600 0.68834900 4.80100200
C -0.95929200 0.09498800 3.97643700

H -0.99552100 -0.59717000 4.81534100
H -1.08381400 1.12388700 4.31279700
C -1.77694000 -0.25126600 2.82112800
H -2.52262800 0.47402100 2.50343200
H -2.12942000 -1.28207000 2.74610900

1INT1

N -0.20660100 -0.19737800 -1.17795500
P 1.16681300 -0.03241600 -0.12867000
P -1.65069100 -0.16235600 -0.21552800
Cr -0.26140400 0.39473000 1.88846800
C 0.93785100 1.10927500 3.39042700
C 1.47855500 -0.10602300 4.15327300
C -0.15983300 -1.42954600 2.81543200
C 0.35918200 -1.15566000 4.23246400
C -0.16373700 0.10221600 -2.62317100
C 2.08869600 -1.60404300 -0.21531900
C 2.21575600 1.31062400 -0.78529500
C -2.51827800 1.38866800 -0.66243300
C -2.72142000 -1.51267600 -0.81282800
C 3.30828400 -1.71783600 0.48726100
C 4.00838500 -2.92833600 0.48656700
C 3.48613600 -4.02759700 -0.19926400
C 1.57777500 -2.72334200 -0.87867000
C 2.27230700 -3.93391100 -0.87872200
O 3.71411800 -0.59265600 1.13894500
C 2.21740100 2.52869300 -0.09035100
C 3.00151500 3.60456300 -0.50333400
C 3.80877100 3.46023200 -1.63066900
C 3.04495200 1.17952400 -1.92657500
C 3.83607200 2.25890000 -2.34009200
O 3.00990800 -0.01545100 -2.56990300
C -2.22862500 2.55373500 0.07771200
C -2.85855000 3.76204700 -0.22675000
C -3.78809600 3.81260100 -1.26937600
C -3.46206600 1.46872500 -1.69434100
C -4.09663200 2.67103200 -2.00512200
O -1.30293000 2.44019300 1.11704200
C -2.37215400 -2.39979000 -1.83774500
C -3.20681400 -3.46553100 -2.17949900
C -4.40004900 -3.65312600 -1.48396600
C -3.92770700 -1.72662100 -0.10678700
C -4.76588200 -2.79241300 -0.44627000
O -4.17703500 -0.83462400 0.89285300
C 4.94853400 -0.61193200 1.85287000
C 3.89731200 -0.24523600 -3.66330200
C -0.98112200 3.64358700 1.84375600
C -5.35482200 -0.99278100 1.68312000
H 1.71319300 1.83917100 3.12336400
H 2.32918800 -0.52843400 3.60131200
H 1.84911500 0.15693100 5.15561700
H -1.15850600 -1.88821600 2.78273600
H -0.45395700 -0.76054500 4.86046100
H 0.70087100 -2.07937200 4.72360500
H 0.18230700 1.63218800 4.01520800
H 0.53281700 -2.05365400 2.23745700
H 4.94763400 -3.02352700 1.01860100
H 4.03472000 -4.96504000 -0.19019800
H 0.62592800 -2.64466200 -1.39254600
H 1.86591600 -4.79440900 -1.40081100
H 1.61039500 2.62197300 0.80414500
H 2.99173300 4.53639000 0.05317700
H 4.42969400 4.28569400 -1.96686600
H 4.47079900 2.16991400 -3.21357900
H -2.64768800 4.66477200 0.33196600
H -4.27344500 4.75743100 -1.49525300
H -3.70848400 0.56940800 -2.25127300
H -4.82380600 2.71342400 -2.80977400
H -1.43915700 -2.26240600 -2.37266600
H -2.92448400 -4.14155800 -2.98023900
H -5.05639500 -4.47933700 -1.74106200
H -5.69288300 -2.95967600 0.08914900
H 0.54724200 -0.55803000 -3.12492200
H -1.15590700 -0.04948500 -3.05246400
H 0.13269900 1.14120400 -2.80401400
H 4.92390200 -1.34551800 2.66697100
H 5.78773600 -0.83125500 1.18240200
H 5.06486500 0.38945300 2.26811300
H 4.94206700 -0.11708000 -3.35822900
H 3.73004200 -1.28030900 -3.96280000
H 3.67422400 0.42170300 -4.50456900
H -0.24032500 3.35677400 2.58767900
H -0.56094300 4.39439500 1.16805900
H -1.87147300 4.03855100 2.34206100
H -5.35539300 -1.95846300 2.20179300
H -5.33127400 -0.18477300 2.41505000
H -6.25757700 -0.90177300 1.06826800

1INT2

N -0.15576400 0.42423300 -1.26426900
P 1.31393700 0.03636500 -0.45888200
P -1.50520800 0.33169400 -0.16771500
Cr 0.42834500 -0.46706600 1.80055800
C 0.94009800 1.34205700 2.63371600
C 2.40452100 1.26619100 3.07591400
C 2.15854100 -1.18146300 2.70312600
C 2.66187700 -0.11910500 3.68673100
C -1.62337100 -0.88607600 3.30857800
C -0.62569200 -1.45200900 4.01161500
C -0.24940800 0.57944600 -2.72864700
C 1.85740000 -1.53564000 -1.21292400
C 2.50607900 1.34825400 -0.89137000
C -2.57023100 1.74492200 -0.67938400
C -2.49737400 -1.13483700 -0.69944400
C 1.32535300 -2.71780900 -0.65687600
C 1.66362800 -3.96429100 -1.18809100
C 2.52855300 -4.03250200 -2.28397900
C 2.71846700 -1.63321400 -2.31162000
C 3.05518700 -2.87412100 -2.85271400
O 0.45776700 -2.56707700 0.41427300
C 2.04330400 2.60026600 -1.32164200
C 2.92979200 3.64622100 -1.57674600
C 4.29650600 3.44821100 -1.38406400
C 3.88952000 1.17962700 -0.64907600
C 4.78048900 2.22578800 -0.91574200
O 4.26037400 -0.02628700 -0.14136600
C -2.25706200 3.05064300 -0.23219900
C -3.06316100 4.13470100 -0.60070200
C -4.17428900 3.93092800 -1.42008400
C -3.69123200 1.57554700 -1.50481000
C -4.49196400 2.65447900 -1.87951600
O -1.14465500 3.17420000 0.54522100
C -2.08380400 -2.03125500 -1.69127800
C -2.83270100 -3.17073700 -2.00074000
C -4.01415600 -3.42619500 -1.30728500
C -3.68677000 -1.42622800 0.00853900
C -4.44540600 -2.55975500 -0.29913100
O -4.01053000 -0.53373200 0.99175200
C -0.20345300 -3.74151300 0.90607900
C 5.63754600 -0.26902500 0.14252700
C -0.83105400 4.45349000 1.09385300
C -5.28280100 -0.63590600 1.62984800
H 0.72802300 2.15017600 1.92762400
H 3.05756300 1.39602300 2.20241300
H 2.66604200 2.07135300 3.78007900
H 2.01354600 -2.17096200 3.15864400
H 2.11965500 -0.19483200 4.64095700
H 3.72523300 -0.25652200 3.93618400
H 0.26320100 1.45010200 3.49643700
H -2.27472200 -1.46203800 2.65744100
H -1.86491900 0.16887100 3.40862400
H -0.41765300 -2.51730500 3.95750300
H 2.85678600 -1.29854900 1.86280300
H -0.01712100 -0.87851000 4.70335700
H 1.27068100 -4.88048000 -0.76512000
H 2.78755100 -5.00607400 -2.68966100
H 3.13252000 -0.72546800 -2.74018300
H 3.72347700 -2.93475200 -3.70581900
H 0.97629600 2.75811100 -1.43655000
H 2.55525600 4.60497000 -1.92106400
H 4.99828800 4.25274300 -1.58389000
H 5.84342900 2.09871800 -0.74883500
H -2.83133700 5.13564300 -0.25751300
H -4.78933000 4.78192000 -1.69860800
H -3.94322300 0.58164400 -1.85953100
H -5.35238100 2.49659700 -2.52219200
H -1.16354000 -1.84297900 -2.23106100
H -2.49890700 -3.84228500 -2.78592800
H -4.61041600 -4.30253200 -1.54452400
H -5.36387000 -2.77266000 0.23558000
H 0.39165200 1.39579000 -3.07254000
H 0.05190500 -0.33569200 -3.25084200
H -1.27976400 0.81829500 -2.99997100
H -0.76942700 -4.22455700 0.10457800
H 0.51756700 -4.43866200 1.34473000
H -0.89481600 -3.39689200 1.67426800
H 6.01621600 0.43518800 0.89212100
H 5.68093100 -1.28354000 0.53927800
H 6.24616400 -0.20460300 -0.76690600
H 0.05521000 4.30218700 1.71012700
H -0.60847400 5.18100200 0.30431700
H -1.65107700 4.82772600 1.71762100
H -5.36561100 -1.56165600 2.21208500
H -5.34780100 0.22188600 2.29999500
H -6.09493800 -0.58969700 0.89562000

1TS2

N -0.12169300 0.46633500 -1.29243200
P 1.32970500 0.04227200 -0.45808800
P -1.46924900 0.37479100 -0.20697700
Cr 0.11556400 -0.55628800 1.74835600
C 0.62463500 1.15925400 2.75312000
C 2.06088100 1.00847900 3.24597900
C 1.66463500 -1.47682300 3.10358000
C 2.23106400 -0.34277600 3.95259500
C -1.46141700 -1.28671600 2.96120100
C -0.40428500 -1.86033600 3.69330100
C -0.20807600 0.66492000 -2.75106700
C 1.91284500 -1.46561600 -1.31636900
C 2.54626500 1.36273900 -0.80084900
C -2.48580800 1.85069800 -0.62318800
C -2.51369100 -1.03845900 -0.76655900
C 1.37827800 -2.69858600 -0.88508900
C 1.75453900 -3.88984600 -1.51162500
C 2.66386900 -3.85435300 -2.57226600
C 2.82129300 -1.46094400 -2.38115700
C 3.19931200 -2.64573400 -3.01317800
O 0.47730700 -2.65617900 0.16638400
C 2.15513200 2.61718800 -1.28666400
C 3.08500300 3.64420800 -1.45651400
C 4.42023200 3.42412500 -1.12169100
C 3.89329100 1.17396000 -0.41540200
C 4.82956000 2.19753100 -0.59425800
O 4.17770000 -0.03739500 0.14214900
C -2.06055900 3.13730500 -0.21393900
C -2.83180000 4.26492800 -0.51976400
C -4.01753100 4.12446800 -1.24205100
C -3.67751400 1.74551200 -1.35481800
C -4.44304500 2.86829000 -1.66833500
O -0.87743700 3.20012500 0.45890600
C -2.14214000 -1.90902500 -1.79724600
C -2.92207700 -3.02239500 -2.12287100
C -4.08822000 -3.27845000 -1.40354700
C -3.69230800 -1.32452900 -0.03744100
C -4.47732400 -2.43717900 -0.35793300
O -3.97811100 -0.44735300 0.96552700
C -0.17356200 -3.88106400 0.53058800
C 5.52081200 -0.32442800 0.52753700
C -0.42922100 4.46504700 0.94360300
C -5.18282200 -0.60990900 1.71146000
H 0.47504300 1.99749800 2.06874600
H 2.75224400 1.05416000 2.39383600
H 2.34797700 1.82988500 3.92040400
H 1.82805700 -2.45716600 3.55165700
H 1.72111900 -0.31741300 4.92630700
H 3.29092100 -0.53704900 4.17601700
H -0.08993800 1.24513600 3.58385900
H -2.07242900 -1.91884900 2.31977200
H -1.94713200 -0.38478300 3.32451600
H -0.24357600 -2.93101100 3.61489900
H 2.15402300 -1.51821800 2.11444400
H -0.11766000 -1.43455300 4.64907000
H 1.35627500 -4.84353900 -1.18837900
H 2.95063100 -4.78610900 -3.05101500
H 3.23892500 -0.51486700 -2.71286000
H 3.90343200 -2.62383900 -3.83897200
H 1.10985400 2.79508600 -1.51247700
H 2.76762900 4.60563400 -1.84802900
H 5.15415100 4.21354100 -1.25534000
H 5.86671300 2.05224700 -0.31612600
H -2.51530300 5.25117700 -0.20281300
H -4.60462200 5.00892300 -1.47235700
H -4.01184700 0.76896500 -1.68746500
H -5.36025000 2.75923400 -2.23839100
H -1.23029600 -1.72250800 -2.35182800
H -2.62351200 -3.67455900 -2.93800000
H -4.70639600 -4.13668100 -1.65086500
H -5.38531500 -2.65059200 0.19397100
H 0.08427900 -0.23973700 -3.29593000
H -1.23451200 0.92394600 -3.02038400
H 0.44469500 1.48073400 -3.07288400
H -0.71824100 -4.29500200 -0.32292000
H 0.55050000 -4.60890300 0.91163900
H -0.88260800 -3.62335000 1.31578800
H 5.86560200 0.36555400 1.30651500
H 5.50446500 -1.34122300 0.92109300
H 6.19750100 -0.27697900 -0.33344700
H 0.51124900 4.26866600 1.45865100
H -0.25400800 5.16633700 0.11935700
H -1.15000300 4.89654700 1.64755900
H -5.18542100 -1.55893000 2.26081600
H -5.20687600 0.22021700 2.41790300
H -6.06099500 -0.55948800 1.05754600

1INT3

N 0.13589700 0.70118800 -1.44698100
P 1.47098500 0.45114600 -0.36544000
P -1.33667000 0.33300700 -0.59620700
Cr -0.18246600 -0.62443900 1.39451800
C -0.43718200 0.91683800 2.65215000
C 0.44228200 0.19204500 3.66382200
C -0.26468900 -0.37632600 4.90911500
C -1.59030200 -1.89522100 2.17696700
C -2.10506200 -1.71520600 3.61066300
C 0.22352700 0.69355300 -2.91865900
C 2.43403000 -0.97403200 -1.01180600
C 2.58308800 1.88653700 -0.59061100
C -2.22646000 1.91932000 -0.40075800
C -2.36137000 -0.64195200 -1.75081000
C 2.37302600 -2.20099100 -0.32040100
C 3.10820400 -3.30369200 -0.76421600
C 3.90577200 -3.19049700 -1.90550300
C 3.25568100 -0.88752400 -2.14385400
C 3.98462800 -1.98515900 -2.60025700
O 1.56513500 -2.26797600 0.81160500
C 2.27437200 2.99718800 -1.38436900
C 3.13596400 4.09354700 -1.45838200
C 4.31813100 4.08730600 -0.72114100
C 3.77372600 1.90966400 0.17208200
C 4.64124200 3.00302800 0.09829300
O 3.97960200 0.81660800 0.96137700
C -3.41720900 1.92714500 0.36008700
C -4.09899100 3.12627700 0.58891000
C -3.59111700 4.31860000 0.06642100
C -1.73548900 3.12770500 -0.90361600
C -2.41271000 4.32760100 -0.67804600
O -3.81844100 0.70982600 0.81920600
C -3.43724700 -0.07636800 -2.44885100
C -4.22613500 -0.84450800 -3.30392500
C -3.94163400 -2.20000400 -3.46129500
C -2.08132900 -2.01502100 -1.92822900
C -2.87587000 -2.79057900 -2.77964400
O -1.00717800 -2.50350800 -1.24040600
C 1.80445800 -3.34956600 1.72930000
C 5.16533500 0.75047600 1.74982500
C -5.02293300 0.61744900 1.57725700
C -0.73296800 -3.90026300 -1.32800400
H -0.01102400 1.85076700 2.27814400
H 0.97282900 -0.68617400 3.17489800
H 1.29207800 0.82308700 3.95071900
H 0.48392600 -0.53032600 5.69695500
H -1.47414100 1.05395200 2.96084500
H -1.12000900 -2.89060600 2.07000000
H -2.42927900 -1.86940500 1.46867800
H -2.80124300 -2.53662100 3.84245000
H 3.07293400 -4.24903300 -0.23628100
H 4.47151600 -4.05446200 -2.24172900
H 3.34185900 0.06418700 -2.66011700
H 4.61362400 -1.89635500 -3.48040500
H 1.34978100 3.00956200 -1.94967100
H 2.88257400 4.94200500 -2.08604400
H 4.99714500 4.93345700 -0.77109100
H 5.55824100 3.02180800 0.67524800
H -5.01502700 3.14047200 1.16767800
H -4.12635400 5.24567200 0.25033100
H -0.80984600 3.12714600 -1.46812100
H -2.02143600 5.25751500 -1.07826100
H -3.65935000 0.97745000 -2.31661900
H -5.05398200 -0.38879600 -3.83764700
H -4.55029700 -2.81252500 -4.12024600
H -2.67364500 -3.84528200 -2.92035700
H 1.13980200 1.19591900 -3.23704800
H 0.22864500 -0.32698900 -3.31795100
H -0.62752200 1.23119400 -3.34557900
H 1.21867200 -3.12707100 2.62020400
H 1.47001100 -4.30445500 1.31162800
H 2.86745600 -3.40346000 1.98275000
H 5.20583700 1.57147800 2.47538800
H 5.11720100 -0.20142000 2.28003700
H 6.06155000 0.77137200 1.11892100
H -5.13439100 -0.43784400 1.82717600
H -4.95729700 1.20794800 2.49868400
H -5.88594000 0.94841800 0.98779400
H 0.13640800 -4.07025600 -0.69550400
H -1.57925100 -4.49206200 -0.96096900
H -0.49345600 -4.19260200 -2.35678000
C -1.02393600 -1.69931200 4.70899900
H -1.48657200 -1.95873000 5.66962000
H -0.29815300 -2.50384800 4.50596000
H -0.95463200 0.38927500 5.28704300
H -2.70397200 -0.79640400 3.68107400

1TS3

N -0.01735700 0.33394300 -1.55763700
P 1.29266100 0.39556500 -0.43515100
P -1.41628000 -0.12425800 -0.64540000
Cr -0.09175700 -0.36058400 1.52898000
C -0.66279100 -2.42764200 4.68026200
C 1.58233600 0.01814900 2.75180400
C 0.55586700 -0.39138800 3.68119200
C 0.68367900 -1.72442800 4.43387200
C -1.37922500 -2.80628700 3.37006700
C -1.76922100 -1.60378800 2.49141500
C 0.03943900 0.81259600 -2.94504600
C 2.60173800 -0.67886300 -1.14967500
C 1.95318900 2.09991600 -0.55326400
C -2.04231300 -1.68866500 -1.37054700
C -2.73337000 1.11230700 -0.97507900
C 3.87084700 -0.75027100 -0.52910900
C 4.85601300 -1.60944900 -1.02970600
C 4.57793700 -2.41373100 -2.13683200
C 2.34516000 -1.51429100 -2.24432300
C 3.32410000 -2.37523600 -2.74449100
O 4.04210300 0.05493200 0.55531900
C 3.05903800 2.43841000 -1.34271000
C 3.51508800 3.75412300 -1.42349700
C 2.86001000 4.74913000 -0.70011000
C 1.29506900 3.12313500 0.16528400
C 1.75322300 4.44281900 0.09451200
O 0.20791500 2.73897600 0.90308800
C -3.25360800 -1.81653400 -2.06455300
C -3.69108400 -3.05285400 -2.54175400
C -2.91460900 -4.18714600 -2.31701900
C -1.28108500 -2.86041700 -1.12903100
C -1.71404900 -4.09974600 -1.60823600
O -0.13038600 -2.69439300 -0.40410200
C -3.42140500 1.71078500 0.10797100
C -4.41578300 2.66829700 -0.13112500
C -4.73409000 3.03764400 -1.43772200
C -3.08942500 1.49884500 -2.27879000
C -4.07514000 2.45524600 -2.51865400
O -3.07123400 1.30747900 1.36583700
C 5.31749000 0.10336500 1.19155000
C -0.51028100 3.73502300 1.62848900
C 0.68629700 -3.83665200 -0.13887300
C -3.89324400 1.70485000 2.46286900
H -1.31464600 -1.77633100 5.28171100
H 2.40374200 -0.66674800 2.54749100
H 1.85983000 1.06825900 2.69592500
H 0.12749800 0.41153000 4.29130800
H -0.62152900 -0.76026300 3.05490900
H 1.33777500 -2.39365800 3.85671700
H 1.19556200 -1.53763100 5.38666100
H -0.49321500 -3.33032300 5.27873800
H -2.14978400 -1.94321700 1.52551400
H -2.55625200 -1.00416500 2.96238400
H -2.27877300 -3.39298200 3.60475500
H -0.72349200 -3.47906600 2.79672500
H 5.83311300 -1.65820100 -0.56374300
H 5.35044100 -3.07552900 -2.51776000
H 1.36318200 -1.49756900 -2.70336400
H 3.10805300 -3.00402300 -3.60270600
H 3.57055800 1.65988500 -1.89999800
H 4.37221300 3.99705300 -2.04360100
H 3.20571200 5.77770200 -0.74961200
H 1.25853900 5.23306600 0.64621700
H -3.86869400 -0.93876700 -2.22702900
H -4.63026300 -3.12550600 -3.08087100
H -3.24211100 -5.15562600 -2.68384800
H -1.13266200 -4.99667800 -1.43209800
H -4.94708900 3.12733800 0.69380200
H -5.50795800 3.78133400 -1.60448000
H -2.60264200 1.02996000 -3.12617000
H -4.32924000 2.73242100 -3.53672700
H 1.07874100 0.83181600 -3.28136400
H -0.51508600 0.13500300 -3.60436100
H -0.36858600 1.82449000 -3.04062500
H 6.09468200 0.43547300 0.49352800
H 5.21682700 0.83002400 1.99810000
H 5.59093500 -0.87259700 1.61002500
H -1.33176100 3.20597400 2.10999600
H 0.12381300 4.20798900 2.38757900
H -0.91242600 4.50028600 0.95472400
H 1.53850400 -3.46362800 0.42948100
H 0.14178700 -4.58153100 0.45230000
H 1.04477200 -4.28666000 -1.07054000
H -3.49392100 1.18547800 3.33475800
H -3.84490200 2.78745600 2.63194400
H -4.93524300 1.40549900 2.30382900

1SC2

N 0.14355700 -0.12693000 -1.59757300
P 1.37901100 0.00664400 -0.37833200
P -1.35626300 -0.10597700 -0.74864200
Cr -0.28040600 0.08481200 1.48608100
C -1.63248200 -0.97023400 4.92617700
C 1.35375200 0.42274200 2.84364700
C 0.14851200 0.36280100 3.57055200
C -0.14753900 -0.75776700 4.55916000
C -2.39548300 -1.98266400 4.05713000
C -2.34455900 -1.70240600 2.55332200
C 0.33500400 0.07401300 -3.03935200
C 2.48734500 -1.42981100 -0.67578600
C 2.37711300 1.46234900 -0.87987600
C -2.44056200 -1.38003800 -1.48865300
C -2.19348200 1.49643600 -1.06629600
C 3.67779400 -1.55692600 0.07724600
C 4.50883200 -2.66907600 -0.09716500
C 4.15211300 -3.66481700 -1.00941200
C 2.14541600 -2.45248400 -1.56831300
C 2.97192400 -3.56496300 -1.74375200
O 3.93277300 -0.54220500 0.94825800
C 3.50370800 1.34372600 -1.70450700
C 4.24060100 2.46156700 -2.09438300
C 3.85036700 3.72341500 -1.65015300
C 1.99215400 2.75042300 -0.44484200
C 2.73247900 3.87463400 -0.82774600
O 0.87687300 2.82103600 0.34591400
C -3.62298800 -1.08860600 -2.18444000
C -4.45122200 -2.10323000 -2.66555800
C -4.10338800 -3.43370800 -2.44315700
C -2.11460300 -2.74033100 -1.25435600
C -2.94342100 -3.75835000 -1.73662700
O -0.97510800 -2.96792900 -0.54164400
C -2.58219500 2.27042900 0.04991200
C -3.20811100 3.50829200 -0.12683100
C -3.43286200 3.99473000 -1.41628200
C -2.42740300 2.01844300 -2.34861600
C -3.03824000 3.25875200 -2.53197800
O -2.30338100 1.75731100 1.30564000
C 5.14194700 -0.56255300 1.70344000
C 0.50432300 4.08924600 0.87892400
C -0.62043700 -4.31211000 -0.21857000
C -2.93973900 2.37011900 2.43656800
H -2.15106300 0.00045900 4.89865300
H 2.04908900 -0.41404600 2.86680200
H 1.80032200 1.37733800 2.57556200
H -0.31559500 1.32058500 3.83209800
H -1.33594200 -1.89235100 2.15024900
H 0.28957600 -1.70143800 4.19949200
H 0.39997700 -0.52102800 5.48407600
H -1.69853700 -1.30857800 5.96712800
H -3.01398200 -2.35854500 1.98624100
H -2.64565000 -0.67010400 2.32221200
H -3.44361300 -2.00712000 4.38148600
H -1.99655700 -2.98911100 4.24337500
H 5.42657100 -2.76621000 0.47077500
H 4.80531600 -4.52315300 -1.13790500
H 1.21439900 -2.38074100 -2.11879200
H 2.69623400 -4.34157600 -2.45062300
H 3.81168100 0.35814800 -2.03961500
H 5.10933000 2.34593400 -2.73482500
H 4.41479200 4.60461400 -1.94148600
H 2.44684300 4.86585100 -0.49713600
H -3.90799300 -0.05519000 -2.34381100
H -5.35894500 -1.85282200 -3.20516400
H -4.73886500 -4.23371800 -2.81191800
H -2.69691000 -4.79917200 -1.56473700
H -3.52012700 4.10161300 0.72403300
H -3.91939800 4.95777200 -1.54024400
H -2.13969300 1.43840500 -3.21949400
H -3.21132400 3.63949500 -3.53350800
H 1.29624700 -0.34473500 -3.34822500
H -0.45438200 -0.45042200 -3.58856800
H 0.31997900 1.13629600 -3.31031800
H 6.01870200 -0.55880000 1.04537000
H 5.13117700 0.34874800 2.30188000
H 5.18221900 -1.43519100 2.36621300
H -0.35959400 3.89838500 1.51527500
H 1.31325000 4.51965600 1.48059500
H 0.22204000 4.78807200 0.08320700
H 0.30903300 -4.24410800 0.34659400
H -1.39586200 -4.78895300 0.39236900
H -0.44934700 -4.90519900 -1.12418100
H -2.70139800 1.73788500 3.29090400
H -2.55141900 3.37920300 2.61258600
H -4.02418200 2.40888000 2.29312500

1P

N -0.50955500 1.29203500 0.89594900
P -1.67586000 0.42760400 -0.05737100
P 1.10881000 0.84548100 0.40021400
Cr 0.21650300 -1.06048600 -1.20537200
C 1.19673200 -0.74944200 -3.32307500
C 0.72235700 -2.00993300 -3.52445600
C 3.02354000 -3.28576900 -3.30187400
C 1.51174000 -3.29965600 -3.62620700
C 2.87774800 -3.06347300 -0.72096600
C 3.41106200 -3.84736300 -1.92296000
C -0.78694300 1.86123600 2.22724200
C -2.49728900 -0.76647900 1.08598200
C -3.00035000 1.64057300 -0.43109600
C 1.83403600 2.37698400 -0.31585800
C 2.04279700 0.62777100 1.96665100
C -2.20613000 -2.14107500 0.97168200
C -2.81701600 -3.07301800 1.81522900
C -3.72244500 -2.64036400 2.78694000
C -3.42058600 -0.36724900 2.06171200
C -4.02900200 -1.28750200 2.91515100
O -1.29834200 -2.53723900 -0.00979800
C -2.88963900 3.01353800 -0.18192500
C -3.89271400 3.90476400 -0.56985100
C -5.02171500 3.42253300 -1.22744200
C -4.13938800 1.17482900 -1.12901500
C -5.14908800 2.06108800 -1.51423500
O -4.15401300 -0.16396800 -1.39370800
C 3.10742200 2.28751900 -0.92245500
C 3.67475700 3.39997500 -1.55009200
C 2.96979500 4.60654800 -1.58591500
C 1.14816000 3.59345600 -0.37775900
C 1.70777600 4.70926400 -1.00496600
O 3.71005400 1.06201900 -0.84099200
C 2.87516700 1.62574000 2.49096600
C 3.59925900 1.42594100 3.66651300
C 3.49370200 0.20750100 4.33384400
C 1.94905700 -0.60176800 2.65744800
C 2.67359500 -0.80755400 3.83674600
O 1.12003800 -1.54191100 2.10890100
C -1.33463600 -3.91120700 -0.42255300
C -5.27333500 -0.71680100 -2.07844500
C 5.05192500 0.92728700 -1.30063400
C 0.98769600 -2.79298900 2.77491800
H 0.56300200 0.11494400 -3.51727200
H 1.77717700 -3.07012200 -0.69303300
H -0.32324700 -2.10613600 -3.82654500
H 3.42875900 -2.27285200 -3.41621200
H 1.37520200 -3.62520600 -4.66871200
H 1.01265000 -4.08384000 -3.03654900
H 2.24532800 -0.54102600 -3.12989000
H 3.21806600 -3.50380800 0.22297500
H 3.20525100 -2.01715200 -0.73515100
H 3.07112800 -4.89044400 -1.85557800
H 3.53462100 -3.88996400 -4.06011400
H 4.50631200 -3.88501200 -1.86361000
H -2.60077700 -4.13136600 1.72923200
H -4.18978300 -3.37322900 3.43822400
H -3.68406600 0.68384600 2.13475600
H -4.74051500 -0.95203000 3.66327500
H -2.00650300 3.39400600 0.31754000
H -3.78869000 4.96438100 -0.35834600
H -5.81040900 4.10427100 -1.53213800
H -6.02688400 1.70435400 -2.04009400
H 4.65352800 3.33796800 -2.01136900
H 3.41715700 5.46604100 -2.07677100
H 0.16153200 3.66274300 0.06654000
H 1.16156000 5.64682500 -1.03760200
H 2.95688000 2.57333200 1.96817600
H 4.23572400 2.21475700 4.05508400
H 4.05070700 0.03546400 5.25046000
H 2.60605800 -1.74617400 4.37344000
H -1.79004400 2.29397700 2.24687800
H -0.72038700 1.10262700 3.01580400
H -0.06927400 2.65541800 2.45056800
H -0.72527000 -3.97267800 -1.32529800
H -0.90965500 -4.56947500 0.34259300
H -2.36049300 -4.21830100 -0.64892700
H -5.38095800 -0.28385400 -3.08031600
H -5.07189700 -1.78550400 -2.16422800
H -6.19970200 -0.56604300 -1.51164800
H 5.34176800 -0.10039200 -1.07717900
H 5.12521100 1.10095800 -2.38134500
H 5.72133800 1.61714600 -0.77371300
H 0.29009300 -3.37494200 2.17366200
H 1.94905300 -3.31735700 2.83310800
H 0.57687100 -2.66566500 3.78327300

1INT4

N -0.25646700 0.34578900 -1.37987000
P -1.58049300 0.09275000 -0.28447400
P 1.25776500 0.29864500 -0.55239200
Cr 0.24778900 -0.38944400 1.76074900
C 1.89140600 -0.27282300 2.94372200
C 3.08636400 -1.19001800 2.66138400
C 2.97984000 -2.61080500 3.23771000
C -0.10347900 -2.39861600 1.63895300
C 0.51620000 -3.33696100 2.67185400
C 2.02996200 -3.59576800 2.53653600
C -1.12128000 0.75230000 3.63018100
C -1.59859000 -0.50415800 3.67831800
C -0.36359200 0.63696100 -2.82227700
C -2.76714200 1.44811200 -0.67010300
C -2.46716600 -1.40435200 -0.87735200
C 1.79701400 2.05952800 -0.42106900
C 2.43821800 -0.44837600 -1.74614700
C -2.67924600 2.68135600 0.01688100
C -3.56618400 3.72174300 -0.28269400
C -4.54500900 3.54284200 -1.26226800
C -3.76666500 1.29870500 -1.64245700
C -4.65153900 2.33377300 -1.94594500
O -1.69672200 2.78665100 0.96505500
C -2.00666600 -2.20627400 -1.92690900
C -2.68487000 -3.37115600 -2.29599000
C -3.83243500 -3.74798900 -1.60162800
C -3.61278300 -1.82105400 -0.16272900
C -4.29944800 -2.98110200 -0.53078300
O -3.96868900 -1.02153500 0.88981200
C 0.98383000 3.11262300 -0.85750900
C 1.38065900 4.44351900 -0.71151200
C 2.60067300 4.73294200 -0.10258100
C 3.02741000 2.37236400 0.20375500
C 3.42500000 3.70538300 0.36034900
O 3.76568900 1.30442100 0.60919600
C 2.40270600 -1.83709800 -2.01874500
C 3.31784500 -2.40291000 -2.91461000
C 4.26116100 -1.59640200 -3.55183500
C 3.39212900 0.33536400 -2.41195800
C 4.29922700 -0.22490100 -3.30985300
O 1.43479200 -2.55774100 -1.38604900
C -1.52119200 4.03860900 1.62824800
C -5.21087900 -1.26682500 1.54804700
C 5.05826400 1.52629300 1.17079800
C 1.34575700 -3.96034600 -1.63731500
H 2.17346300 0.78617200 2.80177300
H 3.28232900 -1.24857400 1.58138000
H 3.98851800 -0.73221400 3.09809000
H 3.98375800 -3.05582000 3.21385400
H -1.20090100 -2.45464200 1.65171000
H 0.24218600 -2.62521300 0.62455000
H 0.29485100 -3.00067400 3.69627500
H 0.00035300 -4.30701500 2.57708400
H 2.23724200 -4.59092000 2.95082600
H 2.29117800 -3.65332000 1.46925200
H 2.70794900 -2.54716400 4.30250900
H 1.56023600 -0.38834500 3.98734900
H -1.20795100 -1.23756900 4.37835600
H -2.41565100 -0.81944200 3.03447200
H -0.33298600 1.08659000 4.29972400
H -1.54795000 1.48678600 2.95101200
H -3.50711600 4.66790400 0.24158600
H -5.22702500 4.35889300 -1.48321200
H -3.85636700 0.35094600 -2.16423900
H -5.41402000 2.19405600 -2.70578300
H -1.10290700 -1.92318500 -2.45308700
H -2.32168800 -3.97266100 -3.12372500
H -4.37157200 -4.64784400 -1.88323600
H -5.18606500 -3.29589000 0.00722200
H 0.02626200 2.88969000 -1.31334500
H 0.74512500 5.24358900 -1.07887700
H 2.92223900 5.76367200 0.01638200
H 4.36954800 3.94720200 0.83314400
H 3.30033200 -3.46608500 -3.12082400
H 4.96435700 -2.05055000 -4.24404800
H 3.42212600 1.40353900 -2.22990800
H 5.02533600 0.40552600 -3.81313200
H 0.26977400 1.48664400 -3.09357000
H -0.05766000 -0.22144400 -3.42919600
H -1.39698100 0.89336700 -3.06680000
H -1.29147600 4.83576700 0.91334900
H -2.40977100 4.30746900 2.21134000
H -0.67025500 3.90447400 2.29721200
H -5.32238300 -0.46696500 2.28121100
H -6.04510400 -1.23078600 0.83832000
H -5.20608700 -2.23579100 2.06138700
H 5.46180900 0.53607100 1.38054900

H 5.71048100 2.04814300 0.46107100
H 4.99228100 2.09928200 2.10305200
H 2.25452600 -4.47893000 -1.31164800
H 0.49420600 -4.31079600 -1.05399600
H 1.16711400 -4.16000500 -2.70023700

1TS4

N -0.24583600 0.16532300 -1.56484600
P -1.56050500 0.11768100 -0.45058200
P 1.25692400 0.03525900 -0.71143900
Cr -0.10112400 0.05672500 1.73979300
C 1.53221800 0.52550200 2.90065000
C 2.64476900 -0.47573600 3.23474200
C 2.32466000 -1.58698000 4.26095100
C -0.24635000 -2.06265000 2.32801600
C 0.28653500 -3.03063100 3.38422200
C 1.78704900 -2.92006000 3.70133900
C -1.29143200 -0.74497100 3.63089100
C -1.63263700 0.52283800 3.12026600
C -0.35098000 0.37567000 -3.02102000
C -2.32840200 1.77230800 -0.58481100
C -2.78366200 -1.08006700 -1.09398400
C 2.12730900 1.64270500 -0.95351100
C 2.21643400 -1.16067300 -1.72831600
C -1.74877200 2.80666100 0.18185500
C -2.26042100 4.10564700 0.11341600
C -3.35084000 4.37634600 -0.71804200
C -3.42181800 2.07245600 -1.40506400
C -3.93651700 3.36700600 -1.47902800
O -0.66522700 2.47020700 0.97962000
C -2.47670600 -1.98211000 -2.12071400
C -3.39736300 -2.94741200 -2.53268000
C -4.63906200 -3.02365500 -1.90470600
C -4.03050200 -1.20219500 -0.43663000
C -4.95941800 -2.16043900 -0.85427900
O -4.23146200 -0.34617100 0.60735000
C 1.50179500 2.77450000 -1.49060700
C 2.16082700 4.00483500 -1.55884400
C 3.46190200 4.11493400 -1.07056700
C 3.43503300 1.78325900 -0.43293900
C 4.10191800 3.01155700 -0.50175400
O 3.96239200 0.65707100 0.12388600
C 1.90412900 -2.53591300 -1.63147700
C 2.60332900 -3.47589300 -2.39617100
C 3.61107500 -3.05133100 -3.26471300
C 3.22600000 -0.76585400 -2.61468900
C 3.92407300 -1.69875200 -3.38217000
O 0.89114800 -2.86349600 -0.77349000
C -0.02725000 3.53488200 1.70398200
C -5.50092500 -0.33674200 1.25823300
C 5.29902100 0.68745000 0.61915000
C 0.50838700 -4.23198200 -0.65967800
H 1.96645400 1.33409700 2.28846000
H 3.02137600 -0.93740000 2.31142000
H 3.49444100 0.09933300 3.63637200
H 3.25949300 -1.82481200 4.78362400
H -1.15020700 -2.43065100 1.83469600
H 0.52985600 -1.96978800 1.54574500
H -0.29324400 -2.96077900 4.31483100
H 0.10494000 -4.05135600 3.01555100
H 2.01315000 -3.71171500 4.42727800
H 2.36060600 -3.17314300 2.79710000
H 1.65052200 -1.19914400 5.03935100
H 1.15068600 0.98664900 3.82572800
H -0.57143300 -0.79613900 4.43924700
H -2.03364200 -1.53655000 3.63870600
H -1.21717400 1.40904500 3.59498600
H -2.57296700 0.65419900 2.59110400
H -1.82814000 4.91090200 0.69367300
H -3.73925800 5.38966500 -0.76222600
H -3.87762300 1.27671300 -1.98725300
H -4.78340000 3.58300200 -2.12280700
H -1.50459700 -1.93386900 -2.59648800
H -3.14626400 -3.62796300 -3.34025300
H -5.36720300 -3.76490500 -2.22097600
H -5.92269300 -2.24579500 -0.36544000
H 0.48187000 2.70156000 -1.85124600
H 1.66220800 4.86407700 -1.99702400
H 3.98817700 5.06366800 -1.12357400
H 5.10882900 3.11512800 -0.11483800
H 2.37250800 -4.53188100 -2.32305200
H 4.14820700 -3.79017900 -3.85257000
H 3.46606600 0.28799300 -2.70806300
H 4.70194100 -1.36980300 -4.06408200
H 0.30316900 1.18781600 -3.34924700
H -0.06798500 -0.52825800 -3.57061900
H -1.37852700 0.63998300 -3.28224900
H 0.36605000 4.28238400 1.00921400

H -0.72690100 3.99651100 2.40833300
H 0.79468600 3.07838500 2.25115400
H -5.44197300 0.44880400 2.01236000
H -6.30504600 -0.10383500 0.55089900
H -5.70579900 -1.29751400 1.74531800
H 5.50046900 -0.31863200 0.98752800
H 6.00909800 0.93504400 -0.17847100
H 5.40228000 1.40536500 1.44135200
H 1.33465400 -4.84723400 -0.28444700
H -0.31470400 -4.25160700 0.05512500
H 0.16332700 -4.62806400 -1.62187500

1INT5

N -0.20735200 -1.80582600 0.01805300
P -1.50566400 -0.66677500 -0.15384000
P 1.29785900 -0.93549500 -0.04029800
Cr 0.22561600 1.32382500 0.06911100
C 2.01828500 4.94469600 0.33882800
C 0.63375700 5.15792000 -0.29238100
C 0.58042800 4.90478700 -1.81676100
C 0.25324500 1.75689300 -1.90983300
C -0.87777700 2.75612800 -1.71606200
C -0.68174300 4.17249800 -2.29993600
C 2.57385700 3.50881400 0.27186700
C 1.73134200 2.42549700 0.96857300
C -0.32478600 -3.14249800 0.62859200
C -2.66439900 -1.40270400 -1.36544500
C -2.44818900 -0.66068800 1.42254200
C 2.10628400 -1.40871400 -1.61174600
C 2.35204900 -1.65571200 1.26587500
C -3.81591900 -0.65380600 -1.70212300
C -4.71712700 -1.12992900 -2.65847100
C -4.46672700 -2.34561700 -3.29969900
C -2.42890800 -2.60563500 -2.04111900
C -3.32360200 -3.08341600 -3.00083000
O -3.95252100 0.53313300 -1.04258000
C -3.32478900 -1.69137700 1.78756500
C -4.04088700 -1.65027600 2.98319300
C -3.89290200 -0.55097600 3.82628700
C -2.31840800 0.44693900 2.28618100
C -3.04045000 0.50099500 3.48233300
O -1.45908200 1.47227200 1.90294900
C 1.55626700 -2.34569400 -2.49147700
C 2.17280600 -2.63381200 -3.71055300
C 3.34916300 -1.97074200 -4.05597000
C 3.29423600 -0.73725400 -1.97939100
C 3.91517400 -1.02265600 -3.19942400
O 3.75470500 0.16452400 -1.06905400
C 2.09680700 -1.33169800 2.61713800
C 2.91969100 -1.83693000 3.62981800
C 3.98870000 -2.67251800 3.30171900
C 3.43149100 -2.49897800 0.96719900
C 4.24792100 -3.01087800 1.97456700
O 1.01641800 -0.52945500 2.84846300
C -5.10655500 1.33171700 -1.29552900
C -1.58732500 2.72974300 2.58971700
C 4.97505900 0.85337500 -1.33470200
C 0.74631900 -0.13944700 4.19341900
H 1.97360600 5.25041800 1.39399500
H -0.08245700 4.50501000 0.22533600
H 0.29599300 6.17990800 -0.07941100
H 0.65121700 5.86048600 -2.34968400
H 1.21931400 2.19226800 -2.17251100
H 0.01157600 0.92430400 -2.57236000
H -1.82341800 2.32954000 -2.06673200
H -1.07564100 2.90932600 -0.61337900
H -0.65067500 4.08345000 -3.39312500
H -1.57041800 4.77325400 -2.06025900
H 1.46090800 4.33238300 -2.12990700
H 2.73868800 5.62150000 -0.14185700
H 2.39410800 1.61362800 1.29979100
H 1.26955600 2.83175300 1.88682800
H 3.57159000 3.53865600 0.73724300
H 2.75918100 3.22822200 -0.77409500
H -5.60469500 -0.56314500 -2.91324400
H -5.17132900 -2.70690300 -4.04301000
H -1.53608600 -3.17819600 -1.81883000
H -3.12584900 -4.02281500 -3.50740700
H -3.46418800 -2.52786500 1.10894200
H -4.71295100 -2.46089200 3.24661700
H -4.44754400 -0.49692800 4.75850000
H -2.95419900 1.34904600 4.15051600
H 0.63343800 -2.84644400 -2.22066200
H 1.73671200 -3.36713900 -4.38153400
H 3.83743100 -2.18512600 -5.00221700
H 4.82881000 -0.51643000 -3.48817400
H 2.73697600 -1.58992300 4.66844100
H 4.61927900 -3.05911300 4.09720300

H 3.63359900 -2.75604400 -0.06709000
H 5.07730200 -3.66431900 1.72362300
H -1.28637200 -3.58651800 0.36203900
H 0.46945700 -3.79520500 0.25544500
H -0.25500500 -3.09488300 1.72112000
H -6.02454000 0.79329800 -1.03320900
H -5.00545400 2.21013400 -0.65708900
H -5.14983300 1.64591300 -2.34497200
H -0.96542800 3.43792300 2.04480700
H -2.62942600 3.06312500 2.58084200
H -1.22533200 2.65499200 3.61997600
H 5.14120400 1.50186400 -0.47459000
H 5.81008300 0.14994400 -1.43301600
H 4.89598900 1.46280100 -2.24243300
H -0.14771500 0.47992800 4.14794800
H 0.54712000 -1.01260300 4.82523100
H 1.57869600 0.43836300 4.61138700

1TS5

N 0.28283800 -1.55100400 0.88543700
P 1.54637300 -0.46118800 0.46704800
P -1.24110300 -0.84197500 0.39316600
Cr 0.08346100 1.30943500 -0.53899100
C -3.06495500 4.02864800 -1.72561800
C -1.99441000 5.12147800 -1.57070100
C -1.27972000 5.19995700 -0.19565500
C 0.29575200 2.56921900 1.08768100
C 0.64927400 3.36449200 -0.08663200
C 0.21975000 4.83929300 -0.22746800
C -2.62094600 2.59661200 -1.38094700
C -1.29399200 2.16425500 -2.03389900
C 0.45145200 -2.99835700 1.10434600
C 2.70981600 -0.42155800 1.87562500
C 2.50582500 -1.20489400 -0.91013800
C -2.18428000 -0.65693100 1.96035200
C -2.11307800 -2.19798100 -0.48752400
C 3.86144700 0.38969900 1.74992300
C 4.76713100 0.50289300 2.80863600
C 4.51968300 -0.17485000 4.00465100
C 2.47519800 -1.06797900 3.09507300
C 3.37469000 -0.95412600 4.15664500
O 3.99340200 1.03476100 0.55503400
C 3.33186700 -2.32554500 -0.75224500
C 4.03557700 -2.86477300 -1.82905200
C 3.92664600 -2.26607600 -3.08292700
C 2.41882000 -0.60812400 -2.18291100
C 3.12632600 -1.13569400 -3.26601100
O 1.61610500 0.51945000 -2.30524200
C -1.61243000 -0.89925700 3.21305000
C -2.32369800 -0.66272600 4.39146100
C -3.62347600 -0.16668700 4.31795000
C -3.49799700 -0.13914400 1.90467000
C -4.21621000 0.09829200 3.08046500
O -3.97812300 0.09422000 0.64962200
C -1.87273500 -2.40076300 -1.86522300
C -2.53544300 -3.42338500 -2.55397400
C -3.43557100 -4.24781600 -1.87652000
C -3.02305800 -3.04198200 0.16357400
C -3.68362600 -4.06370600 -0.51772000
O -0.97116000 -1.55823200 -2.45609400
C 5.14566600 1.84490300 0.33417800
C 1.84395300 1.37438700 -3.43712700
C -5.32171800 0.54388100 0.49862500
C -0.72718900 -1.70574000 -3.85178000
H -3.42294900 4.04949400 -2.76436600
H -1.23914900 5.00330900 -2.35979500
H -2.47596300 6.08332200 -1.78303000
H -1.35159500 6.22805600 0.17647100
H -0.64261800 2.80819100 1.59227900
H 1.08421300 2.25915900 1.77061100
H 1.71259100 3.27551800 -0.34244300
H -0.21708100 2.83646200 -1.23328100
H 0.72213800 5.36013000 0.59930000
H 0.65763000 5.25090900 -1.14724500
H -1.80073600 4.58703800 0.55151400
H -3.93206300 4.28051000 -1.09966600
H -1.35119800 1.13436800 -2.41377000
H -1.04534000 2.79075500 -2.89830900
H -3.41617400 1.90412300 -1.68428600
H -2.55860800 2.49058800 -0.28987600
H 5.65463000 1.11718600 2.71418500
H 5.22771300 -0.07982800 4.82268800
H 1.57999700 -1.66592700 3.22004800
H 3.17929200 -1.46964900 5.09152900
H 3.44176700 -2.76847700 0.23336700
H 4.66822300 -3.73532800 -1.68750600
H 4.47177500 -2.67006400 -3.93100300
H 3.06678100 -0.68092500 -4.24768200

H -0.59413100 -1.26802400 3.26252500
H -1.86401800 -0.86245100 5.35433900
H -4.18811100 0.02331700 5.22618200
H -5.22644400 0.48875800 3.04248100
H -2.35799400 -3.58669200 -3.61001000
H -3.94270600 -5.03769000 -2.42324200
H -3.21956700 -2.89117400 1.22034600
H -4.38333300 -4.70584900 0.00789800
H 1.40031900 -3.19076100 1.61027600
H -0.35575200 -3.37233400 1.74023000
H 0.43857000 -3.55625800 0.16130500
H 6.06526000 1.25331700 0.41023700
H 5.04658900 2.23212900 -0.68060200
H 5.18450000 2.68110300 1.04219800
H 1.28541200 2.28858100 -3.24226700
H 2.91011800 1.60090000 -3.53473900
H 1.47537200 0.91557600 -4.36044900
H -5.48930400 0.62354200 -0.57593800
H -6.02933200 -0.17595400 0.92641600
H -5.46845500 1.52536000 0.96525400
H -0.01173400 -0.92566600 -4.10930700
H -0.29231300 -2.68607000 -4.07866900
H -1.64604600 -1.56563500 -4.43324600

1SC3

N -0.81968600 -1.42125900 0.85057800
P -1.79053100 -0.30362700 -0.04212000
P 0.85027400 -1.01203500 0.54108400
Cr 0.12465600 0.86969000 -1.05298900
C -1.26169400 -2.17594700 2.03594400
C -3.13558500 -1.28336500 -0.80601600
C -2.65251500 0.78070700 1.16329000
C 1.51050900 -2.39546500 -0.47835400
C 1.70648000 -1.21050200 2.15348000
C -4.13740400 -0.59962500 -1.53456200
C -5.15710900 -1.31176200 -2.17351800
C -5.17795400 -2.70634900 -2.10442500
C -3.17447000 -2.68251100 -0.76837400
C -4.18898600 -3.39716300 -1.40774200
O -4.02239600 0.75813600 -1.56163400
C -3.78093600 0.35429100 1.87486000
C -4.43312400 1.19548000 2.77657900
C -3.96114700 2.49372600 2.95739700
C -2.18362000 2.09669800 1.36494900
C -2.84402500 2.95176200 2.25385800
O -1.06980200 2.48272900 0.65072600
C 0.71673100 -3.46488200 -0.90422200
C 1.23226400 -4.46222000 -1.73465700
C 2.56041800 -4.38908400 -2.14986100
C 2.85054100 -2.32857100 -0.92214200
C 3.37523000 -3.32731300 -1.74759500
O 3.55816600 -1.24640700 -0.48129800
C 1.56049400 -0.22621200 3.15754200
C 2.24124400 -0.35398300 4.37443400
C 3.05604700 -1.46345000 4.60359800
C 2.52557200 -2.31739100 2.41790500
C 3.19795000 -2.45191900 3.63189200
O 0.72278400 0.81124100 2.86956100
C -5.02493600 1.52430400 -2.22743300
C -0.66270800 3.85294400 0.71657700
C 4.97425400 -1.23977200 -0.65178300
C 0.49679300 1.79151800 3.87911400
H -5.92891100 -0.79155600 -2.72818000
H -5.97337600 -3.24932600 -2.60646900
H -2.40440700 -3.22249500 -0.23036700
H -4.20336700 -4.48133200 -1.35908100
H -4.16708900 -0.64615800 1.70173000
H -5.30493400 0.84444500 3.31955500
H -4.46357600 3.16750500 3.64545300
H -2.50588600 3.97010700 2.40348700
H -0.31701000 -3.51118400 -0.58237200
H 0.60174300 -5.28755400 -2.05010000
H 2.97460700 -5.15909900 -2.79423300
H 4.40538600 -3.28457400 -2.08176500
H 2.14193400 0.40022700 5.14538200
H 3.57712000 -1.54974900 5.55279600
H 2.63532800 -3.08798400 1.66278700
H 3.82358400 -3.31999300 3.81391700
H -2.29741800 -2.49766300 1.90350400
H -0.63963900 -3.06611200 2.16336400
H -1.19926800 -1.56946600 2.94641100
H -6.01131200 1.35580500 -1.78027800
H -4.73737400 2.56748700 -2.09270100
H -5.05817500 1.28926200 -3.29762400
H 0.18413100 3.94583200 0.03717200
H -1.47049800 4.51716800 0.39054800
H -0.34585800 4.12415200 1.72925300
H 5.33184100 -0.34918900 -0.13363800

H 5.42676900 -2.13227700 -0.20425000
H 5.25075900 -1.18023100 -1.71093000
H -0.25219200 2.46972300 3.47292600
H 0.10446300 1.33424800 4.79490500
H 1.41521200 2.34475000 4.10944600
C -0.81279900 1.21435300 -2.94026000
C -0.04314200 2.33927300 -2.57321600
C 1.37095300 2.58387600 -3.08597100
C 2.38945700 1.58290900 -2.50479200
C 3.84350500 1.79675400 -2.97012400
C 4.52079900 3.13367600 -2.61220400
C 4.78646100 3.39430300 -1.11380200
C 3.64178600 4.06392800 -0.33957500
H -1.89579300 1.24447000 -2.87592700
H -0.41699900 0.47775600 -3.64329500
H 2.74060000 3.44052600 -0.30140100
H -0.58276300 3.21480000 -2.20981300
H 1.67662900 3.60144900 -2.81948600
H 1.40796400 2.52720500 -4.18449000
H 2.10226600 0.55922700 -2.79407400
H 2.37396300 1.61710300 -1.39536500
H 3.85580400 1.68595500 -4.06331100
H 4.45761600 0.97768200 -2.57429000
H 3.95430500 3.97404700 -3.03593900
H 5.48438900 3.13935700 -3.13742000
H 5.66792700 4.04199200 -1.02854200
H 5.06316400 2.45044000 -0.62114000
H 3.36605300 5.02005900 -0.80125900
H 3.94017400 4.27066000 0.69472800

1P′

N 1.14277500 1.41830100 0.65063700
P 1.98786200 0.10606800 -0.11548000
P -0.54989000 1.38393900 0.21984100
Cr -0.31688200 -0.88711200 -1.07882600
C -4.47509700 -3.69222700 -0.53852100
C -3.10209800 -3.35640500 -1.15103400
C -3.07672900 -3.38729600 -2.68778400
C -1.60244200 -0.70250800 -3.05017000
C -1.01169800 -1.90235100 -3.31089600
C -1.68081200 -3.25992600 -3.31845200
C -5.61335600 -2.70464600 -0.84875500
C -5.41063600 -1.30309300 -0.26042700
C 1.60982300 2.09855200 1.87279900
C 3.60270800 0.80918900 -0.63213900
C 2.43489800 -1.06451600 1.23849800
C -0.87453600 3.02813700 -0.53329700
C -1.45373800 1.45040900 1.81687800
C 4.57161200 -0.07391300 -1.16308400
C 5.79162900 0.41367400 -1.64025900
C 6.04584700 1.78711800 -1.61679100
C 3.87509300 2.18205700 -0.64948900
C 5.08938500 2.67557000 -1.13163400
O 4.21455500 -1.39102600 -1.18178200
C 3.38429800 -0.76766900 2.22674500
C 3.68345400 -1.66958400 3.24763300
C 3.03325400 -2.90196600 3.28199300
C 1.80018100 -2.32115100 1.28604900
C 2.09624800 -3.23422400 2.30170000
O 0.88026000 -2.62736600 0.28624500
C 0.13447700 3.96732100 -0.76885400
C -0.13616400 5.17427200 -1.41753100
C -1.43392300 5.44446600 -1.84592800
C -2.18120700 3.31002000 -0.99265000
C -2.45946600 4.51789300 -1.63953700
O -3.10617000 2.33372100 -0.75895600
C -1.79321200 0.24110400 2.46417500
C -2.48618100 0.26207500 3.67985100
C -2.84435000 1.48325800 4.25461600
C -1.83506100 2.65853500 2.41513700
C -2.52408100 2.68553500 3.62741900
O -1.40999500 -0.91872000 1.84001100
C 5.14387300 -2.34698500 -1.68138800
C 0.73372300 -4.01481000 -0.04864500
C -4.46663100 2.58471700 -1.10038400
C -1.73208100 -2.15718900 2.46659000
H -4.36320900 -3.76727400 0.55278200
H -2.76172100 -2.37667600 -0.78118600
H -2.37498700 -4.09270800 -0.77811600
H -3.50096400 -4.34418200 -3.01861900
H -2.64150200 -0.62595900 -2.74070800
H -1.11106600 0.23104500 -3.31937100
H -0.00595700 -1.89007600 -3.73646200
H -4.52017000 -0.80802600 -0.66743300
H -1.74920400 -3.54573200 -4.38016400
H -1.00503100 -4.00798100 -2.87923800
H -3.73594800 -2.61316600 -3.09951500
H -4.77095000 -4.69343200 -0.88123900

H -6.27371200 -0.66250600 -0.47515900
H -5.29380500 -1.34404300 0.82994900
H -6.54582800 -3.12398600 -0.45037500
H -5.76124500 -2.63131300 -1.93425600
H 6.53989000 -0.26227700 -2.03690200
H 6.99573500 2.15666000 -1.99201400
H 3.12685100 2.87656600 -0.28616800
H 5.28146700 3.74392600 -1.12675300
H 3.91768700 0.17703900 2.17905100
H 4.42289100 -1.41588300 4.00089400
H 3.25538800 -3.61780700 4.06807600
H 1.60744500 -4.20113200 2.33787900
H 1.14482600 3.74527900 -0.44456800
H 0.66010400 5.89287700 -1.58465400
H -1.65933100 6.37921700 -2.35112700
H -3.46118700 4.74207400 -1.98679100
H -2.75040400 -0.65862900 4.18564900
H -3.38075700 1.48485100 5.19923000
H -1.59137300 3.59211900 1.91697600
H -2.80619700 3.63373900 4.07419200
H 2.68973700 2.25678100 1.82327200
H 1.12909900 3.07690000 1.95827700
H 1.38106700 1.51945800 2.77469100
H 6.06801300 -2.34918900 -1.09128900
H 4.65315500 -3.31684400 -1.58771400
H 5.38149400 -2.15873100 -2.73537700
H 0.21417500 -4.04349200 -1.00646300
H 1.71426400 -4.49163200 -0.14360300
H 0.13575900 -4.54751300 0.69890200
H -5.01974300 1.69919600 -0.78644800
H -4.85097400 3.46378700 -0.56986900
H -4.58690600 2.72784100 -2.18131600
H -1.34571900 -2.92986100 1.80327900
H -1.24995400 -2.24252500 3.44721800
H -2.81624800 -2.27783900 2.57577900

2R

N 0.01684500 -0.23837300 -0.59267400
P 1.45480000 -0.11611200 0.37788000
P -1.41339800 -0.07674900 0.39195600
Cr 0.03440000 -0.49221900 2.53886400
C 0.84202100 0.39893900 4.66352200
C -0.51358700 0.45517000 4.74363600
C -0.70903200 -2.95723500 3.07986600
C 0.62561600 -3.02749000 2.90183900
C 0.01251400 0.04150700 -2.03877600
C 2.58261800 -1.40441100 -0.27115600
C 2.28417100 1.46264900 -0.01535700
C -2.18200700 1.54004500 0.03346000
C -2.57617600 -1.31642800 -0.28975000
C 3.94639600 -1.37187200 0.08885300
C 4.80672100 -2.39524700 -0.27613700
C 4.33199500 -3.49459100 -1.01484500
C 2.12041300 -2.51028400 -0.99590000
C 2.97867200 -3.54613600 -1.37516700
O 5.25362800 -4.43613800 -1.32297600
C 2.02539600 2.57483600 0.81104100
C 2.58825700 3.81330900 0.54895700
C 3.44086400 3.97883100 -0.55758600
C 3.13693700 1.64339900 -1.11548900
C 3.71400100 2.88235400 -1.39085000
O 3.94612100 5.22053000 -0.72605500
C -2.11095800 2.54652700 1.01684900
C -2.63035200 3.81203700 0.79372000
C -3.23956500 4.11546000 -0.43677400
C -2.79914500 1.85719900 -1.18807500
C -3.32249300 3.12615300 -1.43035500
O -3.71405900 5.37411000 -0.56036800
C -3.96203800 -1.10279700 -0.29796400
C -4.85193600 -2.10497100 -0.68988900
C -4.36252700 -3.35819800 -1.08336900
C -2.10314300 -2.58946900 -0.66916500
C -2.97523500 -3.59066400 -1.06830800
O -5.12749300 -4.39829600 -1.48694300
C 4.84608800 -5.58010000 -2.06710900
C 4.83265000 5.46444600 -1.81542400
C -4.35108300 5.75879300 -1.77636500
C -6.54305500 -4.23868200 -1.52086500
H 1.40064000 -0.44359200 5.06470100
H -1.08979500 -0.34114800 5.20834600
H -1.06350500 1.35320600 4.47128700
H -1.14627600 -2.86600000 4.07165500
H 1.31503500 -2.99393300 3.74245900
H 1.06650000 -3.20168900 1.92264900
H 1.42850100 1.25011800 4.32455300
H -1.40437100 -3.07350100 2.25135300
H 4.34222600 -0.53163600 0.65315900

H 5.85740700 -2.36966900 -0.00549500
H 1.07681800 -2.56665800 -1.28922100
H 2.58577200 -4.37703400 -1.94962800
H 1.37079800 2.46461500 1.67292300
H 2.39497900 4.67078900 1.18537900
H 3.37218100 0.80469200 -1.76395800
H 4.37144200 2.98366600 -2.24649200
H -1.64508900 2.32800000 1.97520500
H -2.58410200 4.58660200 1.55239200
H -2.89065300 1.10156000 -1.96258200
H -3.79250600 3.33391100 -2.38446700
H -4.36648100 -0.14034100 -0.00028900
H -5.91559800 -1.89734100 -0.68778500
H -1.03650800 -2.79340200 -0.65927500
H -2.61193400 -4.56640200 -1.37502700
H 0.92699700 -0.35157300 -2.49088700
H -0.83926900 -0.46366500 -2.50453700
H -0.04998700 1.11629400 -2.24538800
H 5.74291900 -6.18824000 -2.18925400
H 4.08524000 -6.15685500 -1.52665200
H 4.46020400 -5.29728300 -3.05433500
H 5.10676600 6.51724500 -1.74211200
H 5.73510600 4.84589500 -1.73971000
H 4.34022500 5.28145300 -2.77819600
H -4.64215500 6.80058900 -1.63927200
H -3.66406100 5.68059400 -2.62762600
H -5.24485300 5.15232000 -1.96655400
H -6.93955200 -5.19264600 -1.86981300
H -6.94331600 -4.02000400 -0.52327700
H -6.83783100 -3.44426400 -2.21745500

2SC1

N -0.00303600 -0.09398700 -0.79409400
P 1.36529400 -0.16475000 0.25269200
P -1.36261000 0.08276900 0.25038300
Cr 0.00002100 -0.05967300 2.36062000
C -0.00268200 -0.08742300 -2.26177500
C 2.32158400 -1.64743700 -0.19736500
C 2.42029200 1.26583300 -0.15966300
C -2.24021800 1.61946200 -0.18475200
C -2.49408500 -1.28176400 -0.18195000
C 2.09780400 -2.83997000 0.52242200
C 2.73774900 -4.01881600 0.17613000
C 3.62989800 -4.04376500 -0.91230900
C 3.21514700 -1.68722800 -1.28037400
C 3.86571300 -2.86668400 -1.64083300
C 1.82995400 2.46472000 -0.60912200
C 2.59452900 3.60175200 -0.81831800
C 3.97892800 3.58347800 -0.56856600
C 3.80012500 1.26286800 0.09656400
C 4.57900100 2.40328400 -0.10525400
C -2.01555600 2.77507500 0.59202100
C -2.60001400 3.98757000 0.26241800
C -3.43461200 4.08396800 -0.86640600
C -3.07595500 1.73104600 -1.30802800
C -3.66971900 2.94399600 -1.65275100
C -1.96960700 -2.51623100 -0.61742700
C -2.79884300 -3.60428500 -0.83991300
C -4.18415400 -3.49976200 -0.61710200
C -3.87593600 -1.19202700 0.04539300
C -4.71940400 -2.28302700 -0.16902600
H 2.57405900 -4.93647500 0.73164600
H 3.42051200 -0.78466700 -1.84817400
H 4.54988700 -2.86006900 -2.48120600
H 0.76154500 2.50732200 -0.80015300
H 2.14663900 4.52305900 -1.17665000
H 5.64279200 2.36018400 0.09666400
H -2.43536800 4.87712700 0.86163200
H -3.27967200 0.85893800 -1.92230700
H -4.31003700 2.99276200 -2.52566500
H -0.90238500 -2.62172100 -0.79089600
H -2.40210600 -4.55246300 -1.18831700
H -5.78238800 -2.17359100 0.01084600
H 0.09937800 0.92628200 -2.66521500
H 0.82529300 -0.70099400 -2.62983700
H -0.93463800 -0.52605600 -2.62946400
C 2.09930400 -0.03799100 3.18061000
H 2.58589400 -0.96173000 2.87621600
H 2.53917700 0.87687800 2.78975800
C 1.30123000 -0.00611800 4.30765600
H 1.15252200 -0.89951000 4.90489700
H 1.10653900 0.93397900 4.81426200
C -1.30626100 -0.14624600 4.30423900
H -1.11174900 -1.09403300 4.79631900
H -1.15923700 0.73780200 4.91566300
C -2.10165500 -0.09758400 3.17606200
H -2.58870100 0.83029900 2.88499600
H -2.53976500 -1.00617800 2.76923800

H -1.37454100 2.72352100 1.46955000
H 4.28856300 0.35937300 0.44982900
H -4.31422500 -0.25832700 0.38543200
H 1.41328300 -2.84334000 1.36807500
O -3.95808600 5.30479100 -1.10321300
O 4.63258600 4.74257500 -0.80474700
O 4.20469400 -5.23712400 -1.16855600
O -4.90314800 -4.61719500 -0.86309900
C 6.03723300 4.80303800 -0.56978100
H 6.57812500 4.09330000 -1.20753200
H 6.33534000 5.82032000 -0.82495400
H 6.27341700 4.60647500 0.48308900
C -4.82322100 5.48421400 -2.22320900
H -5.11627900 6.53409200 -2.20277200
H -4.30421700 5.26778200 -3.16464700
H -5.71635600 4.85356900 -2.13966600
C -6.31307500 -4.59075600 -0.65468900
H -6.79748500 -3.85208200 -1.30485300
H -6.66791400 -5.58903500 -0.91229200
H -6.55641300 -4.37558100 0.39287500
C 5.12871400 -5.34474600 -2.25011700
H 5.45473600 -6.38503600 -2.25554800
H 4.64913900 -5.10682300 -3.20713500
H 5.99550400 -4.69062900 -2.09784400

2TS1

N -0.00281300 -0.09150800 -0.83476600
P 1.36795600 -0.16040300 0.21083900
P -1.36508800 0.08092000 0.20909900
Cr 0.00071800 -0.06135700 2.30894600
C -0.00481100 -0.08651500 -2.30299700
C 2.35035000 -1.61629600 -0.26044200
C 2.40397900 1.29501700 -0.15241100
C -2.27044700 1.59244100 -0.24743400
C -2.47739100 -1.31106100 -0.17580000
C 2.12453000 -2.83476900 0.41374700
C 2.78949900 -3.99229800 0.04424500
C 3.70957100 -3.96849300 -1.02086700
C 3.27204700 -1.60665400 -1.32021200
C 3.94807800 -2.76442800 -1.70292500
C 1.81375700 2.48281500 -0.62883700
C 2.56708500 3.63408100 -0.79906200
C 3.93725600 3.64018700 -0.48112700
C 3.76900800 1.31580300 0.17270500
C 4.53548700 2.47012900 0.01068200
C -2.03751200 2.77586900 0.48363800
C -2.64644300 3.96914300 0.12989100
C -3.51476500 4.01715000 -0.97648100
C -3.14004200 1.65502700 -1.34852200
C -3.75885100 2.84838900 -1.71638600
C -1.94765600 -2.53474400 -0.63306500
C -2.76477900 -3.63909900 -0.81852200
C -4.14034100 -3.56106300 -0.53458700
C -3.84905700 -1.24721600 0.11335400
C -4.67919600 -2.35436700 -0.06310300
H 2.62520200 -4.93032900 0.56441800
H 3.47900900 -0.68227300 -1.85110200
H 4.65374600 -2.72024600 -2.52413900
H 0.75539600 2.50609400 -0.87157400
H 2.12083800 4.54768600 -1.17851600
H 5.58848000 2.44666300 0.26552800
H -2.47627700 4.88051200 0.69373100
H -3.35042200 0.75971700 -1.92601000
H -4.42510800 2.85981600 -2.57097700
H -0.88710200 -2.61971100 -0.85229700
H -2.36550200 -4.57972400 -1.18405300
H -5.73501800 -2.26609800 0.16431400
H 0.83662500 -0.68145200 -2.67100200
H -0.92726100 -0.54607900 -2.66928800
H 0.07355500 0.92869200 -2.70782900
C 1.79531500 -0.24004600 3.29920500
H 2.34899700 -1.12689000 2.99221800
H 2.38830000 0.67461700 3.23551400
C 0.91613700 -0.38271700 4.45700700
H 0.78105500 -1.41396000 4.78242100
H 1.13639300 0.26710300 5.30131800
C -0.92027800 0.21053200 4.46215300
H -1.14310700 -0.46018200 5.28929400
H -0.78539800 1.23320900 4.81358400
C -1.79612100 0.09726100 3.29876400
H -2.34772000 0.99209400 3.01162300
H -2.39026300 -0.81454600 3.21060200
H 4.25435500 0.42077000 0.55123500
H -1.37249400 2.76076100 1.34392500
H -4.28880300 -0.32206100 0.47449600
H 1.42064600 -2.87456200 1.24183100
O 4.58096300 4.81136300 -0.68350300
O -4.06011300 5.22304900 -1.23890200

O -4.84801200 -4.69248000 -0.74820400
O 4.30717200 -5.14487700 -1.30204500
C 5.97091100 4.89646800 -0.37950400
H 6.55530600 4.19755200 -0.99026800
H 6.26301600 5.91932900 -0.61889000
H 6.15845000 4.70242700 0.68358900
C -4.95993100 5.35392200 -2.33807600
H -5.26503700 6.40054500 -2.34494400
H -4.46623900 5.11077100 -3.28657400
H -5.84245700 4.71660300 -2.20634800
C -6.24751400 -4.69286000 -0.47773200
H -6.77430800 -3.96411000 -1.10577200
H -6.59422200 -5.69795500 -0.71946800
H -6.44851900 -4.48156700 0.57957000
C 5.26023600 -5.20362800 -2.36194100
H 5.60036500 -6.23891000 -2.39410900
H 4.80239800 -4.93910900 -3.32261300
H 6.11381000 -4.54415600 -2.16476700

2INT1

N -0.15196500 -0.36722900 -0.70002300
P 1.32147000 0.02080400 0.10734400
P -1.48322400 -0.35403700 0.43580200
Cr 0.40994200 -0.48096400 2.34301600
C 1.91619600 -0.17041200 3.67547800
C 2.53257900 -1.54266100 3.97890300
C 0.73663300 -2.49233700 2.52575300
C 1.42643700 -2.60890600 3.88799100
C -0.33317400 -0.20578400 -2.15340000
C 2.60364600 -1.01254900 -0.65457100
C 1.80401200 1.74420200 -0.20109900
C -2.46848900 1.15440200 0.16020400
C -2.54101000 -1.74199600 -0.09776600
C 3.94660800 -0.60369900 -0.64285100
C 4.95905200 -1.43319000 -1.12265100
C 4.63948900 -2.70361300 -1.62666700
C 2.29966400 -2.29772000 -1.14914700
C 3.29655100 -3.12601300 -1.63712000
C 1.68711500 2.68437100 0.83303900
C 1.99543400 4.02942300 0.63500400
C 2.42734600 4.45924300 -0.62920000
C 2.24946100 2.19150300 -1.46445700
C 2.55189200 3.52540200 -1.67695400
C -2.44916300 2.15502600 1.15252400
C -3.13578100 3.34761000 0.98787500
C -3.86642700 3.58178200 -0.19072800
C -3.21132400 1.39944700 -1.00743700
C -3.90179900 2.59568400 -1.19103300
C -1.95416400 -2.97075100 -0.46505200
C -2.73446000 -4.07545500 -0.76370700
C -4.13720500 -3.99623000 -0.68199300
C -3.93929500 -1.67947900 -0.00759800
C -4.73600700 -2.78866900 -0.29608600
H 2.65268200 0.61305000 3.46241000
H 3.30621800 -1.76784400 3.23190000
H 3.02678500 -1.56913700 4.96066200
H -0.22451400 -3.01713400 2.44323600
H 0.69670600 -2.44405800 4.69631900
H 1.82306300 -3.62351800 4.04471400
H 1.29850000 0.17433600 4.53428700
H 1.39574400 -2.78374600 1.69868800
H 5.98405200 -1.08251600 -1.10336600
H 1.27081100 -2.64276800 -1.16604000
H 3.06787100 -4.10997600 -2.03337400
H 1.36684800 2.36770700 1.82341100
H 1.90415600 4.72534200 1.46039900
H 2.89559400 3.87882100 -2.64369700
H -3.13115400 4.11510600 1.75494800
H -3.26964300 0.64229500 -1.78373100
H -4.46420200 2.75007100 -2.10441300
H -0.87360300 -3.05931400 -0.52130700
H -2.28676100 -5.01885600 -1.05917700
H -5.81326500 -2.69974100 -0.21951100
H 0.56826100 -0.54090500 -2.67389300
H -1.16676000 -0.83296200 -2.48171100
H -0.53744500 0.83698100 -2.42333700
H -4.42652200 -0.75574700 0.28797400
H -1.89612500 1.99256500 2.07558200
H 2.37318300 1.48594400 -2.28063800
H 4.21582900 0.37678800 -0.26209400
O -4.80629900 -5.12897900 -0.99025700
O -4.49798100 4.77271500 -0.26086200
O 2.75237200 5.73020400 -0.94140800
O 5.53291400 -3.58539200 -2.12048200
C -5.27422700 5.07967300 -1.41756200
H -5.68226000 6.07533000 -1.24200900
H -4.65289200 5.09327100 -2.32109300
H -6.09632300 4.36531800 -1.54497600

C -6.22978000 -5.13138700 -0.91565300
H -6.53813900 -6.13811100 -1.19894700
H -6.57487600 -4.91654000 0.10296900
H -6.66586900 -4.40601700 -1.61332400
C 2.66966800 6.73604800 0.06706800
H 3.34468400 6.51588000 0.90263100
H 2.97941400 7.66282100 -0.41624000
H 1.64319400 6.84202100 0.43805500
C 6.91608800 -3.23606900 -2.13636800
H 7.43091500 -4.09427400 -2.56871700
H 7.09445900 -2.35093600 -2.75862100
H 7.29140500 -3.05708100 -1.12175600

2INT2

N 0.09350600 -0.05758100 -0.89945800
P -1.29863500 0.23930800 0.07324100
P 1.49169600 -0.30260800 0.09749500
Cr -0.07615200 -0.01131500 2.26697700
C -0.71295800 -1.93147800 2.65692300
C -2.08152300 -1.82987300 3.32942100
C -1.60656600 0.61606600 3.53319100
C -2.07643700 -0.63344700 4.29383900
C 2.21223200 0.01020800 3.51382800
C 1.33606600 0.52041400 4.40032100
C 0.11475500 0.04765200 -2.36704400
C -1.97485300 1.87818400 -0.33924400
C -2.54727100 -0.96965500 -0.46586400
C 2.34423600 -1.78854700 -0.51849200
C 2.62057200 1.09365800 -0.25763200
C -1.70614000 2.96277500 0.52176400
C -2.13838200 4.24389500 0.21827100
C -2.85947600 4.48292800 -0.96614300
C -2.70199400 2.13067300 -1.51442700
C -3.14153700 3.41390000 -1.83306700
C -2.15023400 -2.23111300 -0.95375000
C -3.09075200 -3.19821400 -1.27092200
C -4.46244100 -2.94187400 -1.09088100
C -3.91725600 -0.72675300 -0.28263400
C -4.87154000 -1.69632600 -0.59060700
C 2.15288800 -3.00048500 0.16398000
C 2.73308800 -4.18815500 -0.28055300
C 3.52788600 -4.17948500 -1.43712600
C 3.15323800 -1.79715400 -1.67644400
C 3.73285000 -2.97055400 -2.12988600
C 2.09438300 2.36978800 -0.54584700
C 2.92506600 3.47133000 -0.68396800
C 4.31600900 3.33803700 -0.52153100
C 4.00862000 0.97606000 -0.08688000
C 4.85451900 2.07919800 -0.21751500
H -0.70253500 -2.54796900 1.75093400
H -2.85170200 -1.67177800 2.56266000
H -2.34563000 -2.76525100 3.84632100
H -1.33503100 1.44598200 4.19984100
H -1.39298000 -0.85142900 5.12805000
H -3.06827400 -0.48957100 4.74705700
H 0.06721100 -2.28721700 3.34998400
H 2.81207100 0.65067800 2.87166300
H 2.40921500 -1.05780600 3.45786500
H 1.19173900 1.59276600 4.50366100
H -2.40895500 0.98112700 2.87496300
H 0.78683500 -0.11126500 5.09194400
H -1.94264700 5.07943800 0.88234700
H -2.94307200 1.31549600 -2.19025300
H -3.70067300 3.57200400 -2.74776300
H -1.09589200 -2.45059000 -1.09507500
H -2.79350700 -4.16623800 -1.66113700
H -5.92116700 -1.47240600 -0.44138100
H 2.56694500 -5.10322600 0.27556600
H 3.34156200 -0.87387800 -2.21600700
H 4.35557500 -2.98506200 -3.01858300
H 1.02351900 2.50228700 -0.67266500
H 2.52448500 4.45166600 -0.92158500
H 5.92219700 1.94547300 -0.08858100
H -0.85932200 -0.24395300 -2.76911700
H 0.34933200 1.06522800 -2.69879700
H 0.86202300 -0.64063400 -2.77180600
H 1.54017300 -3.02441000 1.06108300
H 4.45107700 0.00983300 0.13868000
H -1.16541800 2.79828900 1.45090300
H -4.25619800 0.23219200 0.09827000
O 4.13965600 -5.26211900 -1.96099100
O -5.29571200 -3.95043900 -1.42812500
O -3.23628900 5.76167900 -1.17272100
O 5.03654000 4.47137900 -0.67808100
C 3.98870300 -6.52456200 -1.31467400
H 4.56354200 -7.23182900 -1.91293500
H 4.38930700 -6.49844700 -0.29421200
H 2.93728300 -6.83531500 -1.29121300

C -6.70043800 -3.76989900 -1.26492400
H -7.07000200 -2.94455400 -1.88553800
H -7.15761900 -4.70372600 -1.59320200
H -6.95830900 -3.58600500 -0.21495500
C -3.98342700 6.08547000 -2.34418000
H -4.16937000 7.15815200 -2.28670800
H -3.41297600 5.86144700 -3.25350800
H -4.93961900 5.54923300 -2.36558000
C 6.45336000 4.41268400 -0.53496100
H 6.90067700 3.73968300 -1.27656900
H 6.80952400 5.42900500 -0.70562700
H 6.73876900 4.09061700 0.47401200

2TS2

N 0.06826100 -0.20991400 -0.84492600
P -1.39385000 -0.09466200 0.06492600
P 1.45254700 -0.08307000 0.19448900
Cr -0.26642700 -0.04272000 2.35590700
C -1.95415900 0.29918000 3.48943600
C -1.62425600 1.53394400 4.33450200
C 0.78150900 0.92250900 3.96753400
C -0.24945000 1.36051100 4.99793400
C 0.90396600 -1.39930400 3.93613100
C 0.12628600 -2.06491600 2.98951600
C 0.13859500 -0.24338400 -2.31404900
C -2.17519000 1.50110300 -0.35894800
C -2.52507800 -1.38290600 -0.54035100
C 2.43080200 -1.59261600 -0.14525200
C 2.48377200 1.29162600 -0.42246200
C -3.54836800 1.71885900 -0.11914100
C -4.11679400 2.96690900 -0.31953000
C -3.32810400 4.04466000 -0.76224800
C -1.39614700 2.58680900 -0.78318400
C -1.95893000 3.84684500 -0.99311800
O -3.98063000 5.21654300 -0.93291000
C -2.54840300 -2.62703400 0.12362900
C -3.35357800 -3.66174600 -0.32313600
C -4.17029800 -3.48287500 -1.45530300
C -3.34739000 -1.21654600 -1.66695800
C -4.16338400 -2.24935900 -2.12639700
O -4.92069600 -4.54763400 -1.80768200
C 1.80448600 -2.77902200 -0.55338300
C 2.52191100 -3.96835400 -0.69989600
C 3.89546800 -3.99124400 -0.41854500
C 3.81070500 -1.63517900 0.14379700
C 4.53171200 -2.81155600 0.00885300
O 4.69020100 -5.08053200 -0.52527200
C 2.43087800 2.52938300 0.25150900
C 3.15382400 3.62463100 -0.19516700
C 3.96306900 3.51510300 -1.34034100
C 3.30114400 1.19727600 -1.56111500
C 4.03406200 2.28922300 -2.02243200
O 4.62958700 4.63430900 -1.69549700
C -3.24926100 6.35475100 -1.37881100
C -5.79231500 -4.44108100 -2.93179600
C 4.11801300 -6.31065400 -0.95969800
C 5.49044700 4.59667700 -2.83210100
H -2.84074800 0.44557300 2.86074800
H -1.60058700 2.43135700 3.69658300
H -2.39618200 1.72693200 5.09365100
H 1.79751200 0.83678100 4.35446100
H -0.31173000 0.61208900 5.80087100
H 0.07664000 2.29303600 5.48264900
H -2.12457200 -0.58185700 4.12360200
H 0.60912700 -2.51651800 2.12496600
H -0.84876200 -2.45534300 3.26898100
H 1.97659900 -1.32734400 3.79016400
H 0.83116800 1.65245900 3.12095800
H 0.55865800 -1.28899300 4.95808500
H -4.18086400 0.90262500 0.21796500
H -5.17387500 3.13806500 -0.14354600
H -0.33242500 2.45788300 -0.96055800
H -1.32782000 4.65877000 -1.33514500
H -1.93286500 -2.77952000 1.00567200
H -3.38279600 -4.61821300 0.18864500
H -3.36778800 -0.26491400 -2.18980300
H -4.78831500 -2.08543500 -2.99641300
H 0.74044100 -2.78162000 -0.77440800
H 2.00628300 -4.86110500 -1.03423100
H 4.32978900 -0.73655300 0.46556000
H 5.59524300 -2.84660400 0.22220900
H 1.81885800 2.63528400 1.14413100
H 3.12151600 4.57554900 0.32671300
H 3.38387000 0.25443800 -2.09365000
H 4.65530500 2.17807700 -2.90356400
H 0.25115700 0.75981400 -2.74030900
H 0.98643900 -0.85942900 -2.62706200
H -0.77234300 -0.69859500 -2.71257900

```
H -3.97541600 7.16563500 -1.44312900
H -2.46133500 6.62654100 -0.66556000
H -2.80675600 6.18172700 -2.36753900
H -6.28798800 -5.40880000 -3.01210900
H -6.54352800 -3.65717200 -2.77837200
H -5.23107300 -4.24035600 -3.85230900
H 4.93718200 -7.03027000 -0.96980000
H 3.33914700 -6.65436300 -0.26785500
H 3.69921800 -6.22030600 -1.96955000
H 5.91618700 5.59721600 -2.91188600
H 6.29664500 3.86614600 -2.69526100
H 4.93178300 4.36314100 -3.74648600
```

2INT3

```
N -0.06662900 0.22666400 -0.87403000
P 1.08551100 1.01418500 0.16329600
P -1.13435200 -0.80538800 0.00609500
Cr -0.18322400 -0.12946500 2.23188900
C -1.53346300 -0.10903300 3.72056200
C -0.40001400 0.44434900 4.56276400
C 0.54796900 -1.88856400 5.23106500
C 0.16353900 -0.46350800 5.66743100
C 0.75073000 -1.89607700 2.65290800
C 1.47118200 -1.96987300 3.99948300
C -0.08326500 0.37573600 -2.33871600
C 2.75811700 0.66959000 -0.47137800
C 0.81957600 2.80108100 -0.12534000
C -2.83290000 -0.40512400 -0.50089100
C -0.82569900 -2.51028600 -0.55338200
C 3.27044000 1.24547400 -1.64635900
C 4.54235100 0.92460500 -2.11601000
C 5.33850600 0.01185400 -1.40367800
C 3.57395600 -0.23966600 0.23311200
C 4.84224900 -0.56594000 -0.22132800
O 6.58328600 -0.36750800 -1.76258900
C 1.85499600 3.73166500 0.09881000
C 1.62002800 5.09466200 0.00571900
C 0.33708000 5.57560300 -0.31238000
C -0.45917000 3.29295900 -0.42518400
C -0.70565600 4.66293500 -0.52894300
O 0.21648800 6.92064400 -0.38573000
C -3.56338200 0.53764800 0.25249700
C -4.83747500 0.92518700 -0.12836300
C -5.42810300 0.37531900 -1.28198700
C -3.43598400 -0.94979000 -1.64656800
C -4.71794800 -0.56962500 -2.04002200
O -6.67156800 0.81356100 -1.56728700
C -1.78570400 -3.52205300 -0.34340900
C -1.50657600 -4.84226700 -0.65924400
C -0.25286300 -5.19606500 -1.19119300
C 0.42591800 -2.87667100 -1.06662000
C 0.71571100 -4.20167800 -1.39360500
O -0.08458400 -6.50754500 -1.47047300
C 7.16967400 0.19547800 -2.93450800
C -1.05377700 7.48246300 -0.70164300
C -7.34818300 0.28759500 -2.70825000
C 1.16264100 -6.94541700 -2.00285900
H -2.34517200 0.60449400 3.54145900
H 0.50475900 0.70112000 3.89826600
H -0.63350300 1.43821700 4.96214500
H -0.35550100 -2.48131400 5.03552400
H -0.58526500 -0.52342300 6.46706400
H 1.04397700 0.02826700 6.10196100
H -1.92583500 -1.07649300 4.03555900
H 1.44224300 -2.01913400 1.80745100
H -0.03068500 -2.66274400 2.57162600
H 2.25529100 -1.19931200 4.06793800
H 1.04142200 -2.36852900 6.08437700
H 2.00823100 -2.93061400 4.03828600
H 2.67990000 1.96930500 -2.20045500
H 4.90645900 1.39016000 -3.02441100
H 3.21343500 -0.68764700 1.15492400
H 5.47725500 -1.25785600 0.32235500
H 2.85674300 3.38747900 0.33892400
H 2.41545300 5.81436800 0.17020600
H -1.28071900 2.60247000 -0.59649500
H -1.70313900 5.00473100 -0.77947200
H -3.13080900 0.96212600 1.15497200
H -5.40830000 1.64249500 0.45232000
H -2.90858400 -1.69319000 -2.23664600
H -5.15378300 -1.01303200 -2.92746700
H -2.76232900 -3.27518500 0.06328400
H -2.24269100 -5.62479400 -0.50640900
H 1.19364900 -2.12334800 -1.21781300
H 1.68999400 -4.44677600 -1.79993400
H 0.63476000 -0.29530800 -2.82270700
H -1.08479400 0.15847000 -2.71996200
H 0.15825400 1.40881200 -2.60375900
```

H 8.16212700 -0.24960800 -3.00949900
H 6.58825600 -0.05500400 -3.83003000
H 7.26318100 1.28455600 -2.84757800
H -0.90656400 8.56288800 -0.70413100
H -1.39771000 7.15893600 -1.69185600
H -1.80492800 7.21834600 0.05295600
H -8.32083400 0.77968300 -2.72558800
H -6.80529600 0.51761600 -3.63285400
H -7.48807300 -0.79637900 -2.62114200
H 1.06233700 -8.02224300 -2.14162500
H 1.37166100 -6.47062400 -2.96932600
H 1.98555700 -6.74203400 -1.30673600

2TS3

N -0.08039200 0.17636400 -1.02593900
P 1.29986300 0.43511700 -0.02220200
P -1.37175900 -0.27657800 0.02673600
Cr -0.06142600 0.05452300 2.11645400
C -1.84150700 -0.21961200 3.18041600
C -0.91588100 0.28956900 4.17202500
C 0.83540400 -1.33155700 5.24223500
C -0.49901000 -0.57258100 5.39079300
C 1.83938200 0.00856900 3.31747800
C 1.98477700 -0.41074800 4.79444000
C -0.11440900 0.23523100 -2.49337500
C 2.65636800 -0.62003400 -0.62793500
C 1.83656100 2.15412400 -0.32615000
C -2.82050700 0.72254900 -0.43560000
C -1.81585500 -2.00477400 -0.35447200
C 3.49834300 -0.24642000 -1.68762300
C 4.48477800 -1.10582900 -2.16806200
C 4.64771900 -2.37405600 -1.58729500
C 2.83373000 -1.89814000 -0.05764500
C 3.80992000 -2.76319600 -0.52565600
O 5.56931600 -3.28338800 -1.96843000
C 3.14149300 2.57173300 0.01014100
C 3.51275100 3.90264400 -0.09866300
C 2.58792700 4.86492700 -0.54378100
C 0.91825000 3.12489700 -0.75026900
C 1.28319400 4.46678700 -0.87013900
O 3.05271400 6.13204400 -0.62124500
C -3.03620000 1.95412400 0.21749900
C -4.08632900 2.78213200 -0.14391500
C -4.96260500 2.40116700 -1.17776200
C -3.70249900 0.35463800 -1.46412800
C -4.76404600 1.17795900 -1.83762600
O -5.95545800 3.27282600 -1.45264600
C -3.05558500 -2.53567500 0.05933800
C -3.35295000 -3.87618600 -0.12802000
C -2.41643400 -4.73431100 -0.73365500
C -0.88429500 -2.87251500 -0.94017500
C -1.17526900 -4.22314900 -1.13967600
O -2.80834200 -6.02027500 -0.87650000
C 6.46844400 -2.95756200 -3.02703400
C 2.17408400 7.16320300 -1.06245600
C -6.89484100 2.95308200 -2.47755200
C -1.91652000 -6.95013700 -1.48475600
H -2.65287800 0.42488300 2.84961000
H 0.39229700 0.39101400 3.69215500
H -1.07464700 1.34325600 4.41898200
H 0.72525600 -2.15370500 4.51681000
H -1.30544000 -1.27988600 5.61527900
H -0.41641200 0.09596800 6.25603600
H -2.11341800 -1.27634200 3.22593900
H 2.42052200 0.90168600 3.06937300
H 2.18684600 -0.81584100 2.68034600
H 2.00434700 0.47814600 5.44051600
H 1.07548100 -1.80222000 6.20221200
H 2.94806600 -0.91700000 4.93760400
H 3.39413500 0.73379800 -2.14272800
H 5.11750300 -0.78260200 -2.98633000
H 2.19826300 -2.22054000 0.76343200
H 3.95555600 -3.74395300 -0.08465400
H 3.87740300 1.84835100 0.34966300
H 4.51725100 4.22829600 0.15157000
H -0.09941000 2.83692900 -0.99882800
H 0.55065500 5.18656300 -1.21648800
H -2.37404800 2.26442800 1.02230800
H -4.26219400 3.72659000 0.36075200
H -3.57226700 -0.59351800 -1.97701400
H -5.42732400 0.86048400 -2.63357100
H -3.79884800 -1.89265600 0.52246000
H -4.30765400 -4.28921900 0.18124900
H 0.08648100 -2.49835300 -1.25135400
H -0.43587800 -4.86131800 -1.60921700
H 0.17326700 -0.71966500 -2.94726900
H -1.12466500 0.49450300 -2.82404300
H 0.56694700 1.01455700 -2.84627500

H 7.11197300 -3.82967800 -3.14491100
H 5.92888100 -2.77212400 -3.96351300
H 7.07996600 -2.08350500 -2.77338100
H 2.76285200 8.08076300 -1.04361100
H 1.82210800 6.97619700 -2.08442600
H 1.31372200 7.26784500 -0.39008700
H -7.59079800 3.79163000 -2.51031200
H -6.40044800 2.84979500 -3.45094600
H -7.44202100 2.03272900 -2.24113700
H -2.44437800 -7.90421300 -1.49121400
H -1.67809100 -6.65803700 -2.51486100
H -0.98969200 -7.05000200 -0.90655500

2SC2

N -0.17798800 0.21980000 -0.97603300
P 1.20080700 0.62413000 -0.01523800
P -1.37518100 -0.41620200 0.10074400
Cr -0.02961700 -0.06708100 2.08982500
C -1.77834200 -0.68672300 3.23394500
C -0.68406800 -0.45882300 4.08222600
C 1.61569200 -1.65132500 4.51598700
C 0.08957700 -1.58669800 4.75205700
C 2.38074300 0.66150600 3.68847600
C 2.39835500 -0.36271200 4.82876700
C -0.26994400 0.32706600 -2.43794400
C 2.65108500 -0.25437700 -0.68167900
C 1.51713600 2.39810700 -0.31730800
C -2.93488600 0.44756200 -0.26158000
C -1.64332100 -2.16075100 -0.36330700
C 3.33874700 0.15751800 -1.83517700
C 4.40520000 -0.58011000 -2.34623100
C 4.80897200 -1.76075800 -1.70092000
C 3.07249400 -1.44132200 -0.04694900
C 4.13153200 -2.18488400 -0.54310000
O 5.82692100 -2.55021500 -2.10375700
C 2.79619500 2.95508800 -0.11162400
C 3.00275000 4.32178100 -0.21706300
C 1.93340500 5.18076100 -0.52915400
C 0.45603300 3.26577400 -0.61359800
C 0.65365900 4.64244500 -0.72912900
O 2.24229400 6.49434100 -0.61325400
C -3.25681600 1.60549100 0.47660700
C -4.39776600 2.33945700 0.19595400
C -5.26183000 1.93359200 -0.83865100
C -3.80608000 0.05323400 -1.28939800
C -4.95871200 0.78163300 -1.58155800
O -6.34827100 2.71099700 -1.03160700
C -2.82268900 -2.83696500 0.01388200
C -2.97483400 -4.19285100 -0.23034400
C -1.94766500 -4.92217700 -0.85666800
C -0.62017800 -2.90170500 -0.97101600
C -0.76430100 -4.26640900 -1.22651300
O -2.19883300 -6.23600300 -1.05439400
C 6.57867800 -2.17922500 -3.25796900
C 1.21044500 7.42692200 -0.92271200
C -7.28193500 2.35601500 -2.04996100
C -1.20470300 -7.04071500 -1.68164100
H -2.56326100 0.05882600 3.12355100
H 1.35937200 1.04208500 3.49188300
H -0.64368600 0.50946900 4.58961700
H 1.81980000 -1.94740700 3.47171100
H -0.34512200 -2.54551700 4.44091000
H -0.08158600 -1.51452400 5.83628200
H -2.07730600 -1.70562000 2.98990500
H 2.97145000 1.55504600 3.91858800
H 2.79231900 0.23054700 2.76764400
H 2.01280600 0.09246300 5.75066400
H 2.00809200 -2.47134300 5.12979100
H 3.44553100 -0.61876100 5.03082600
H 3.05099000 1.07472800 -2.34073000
H 4.91435700 -0.23118100 -3.23701600
H 2.56500000 -1.78654800 0.85135500
H 4.46402400 -3.09437300 -0.05332500
H 3.64103500 2.31396900 0.12280800
H 3.98640300 4.75490300 -0.06685600
H -0.54351100 2.86785000 -0.76564700
H -0.18756300 5.28048800 -0.97370900
H -2.60581200 1.93017400 1.28503000
H -4.65525300 3.22609300 0.76625600
H -3.59527100 -0.84259400 -1.86581700
H -5.61056200 0.44552200 -2.37927700
H -3.63417900 -2.29662300 0.49294600
H -3.88300400 -4.71627200 0.05066700
H 0.30839900 -2.41535600 -1.25589900
H 0.04302300 -4.80347900 -1.71066500
H 0.15100300 -0.55202400 -2.93902200
H -1.31903100 0.43270600 -2.72909400
H 0.26489800 1.21929800 -2.77676600

H 7.33636400 -2.95410300 -3.37675200
H 5.94466500 -2.14786200 -4.15220100
H 7.06795400 -1.20805200 -3.11747700
H 1.69053600 8.40587500 -0.93236800
H 0.77355600 7.22501100 -1.90841800
H 0.42146100 7.41475900 -0.16079400
H -8.06239100 3.11628800 -2.01129300
H -6.81154400 2.36481800 -3.04055500
H -7.72200100 1.37007300 -1.85862200
H -1.62675100 -8.04479200 -1.73324200
H -0.98533500 -6.68435400 -2.69563800
H -0.28018500 -7.06327300 -1.09156200

2P

N 0.50528500 -0.09578200 -0.81200100
P -1.10743300 -0.27439800 -0.20425800
P 1.65748100 0.19005700 0.45355700
Cr -0.21753300 -0.13921200 2.24631200
C 0.05159500 -0.31568500 4.54765800
C -1.30628300 -0.42046400 4.41876900
C -3.45669300 0.87544200 3.70915400
C -2.27633400 0.70428800 4.69556200
C -4.47572700 -1.52871300 3.73397200
C -4.68425600 -0.02332600 3.94276100
C 0.83078900 -0.06225100 -2.24739800
C -2.15736300 0.96883600 -1.02990600
C -1.68081800 -1.89016300 -0.84319200
C 3.05662700 -0.93892700 0.15286000
C 2.32126400 1.87213100 0.17584600
C -2.63576900 0.82940700 -2.34276800
C -3.38930100 1.83165200 -2.95147000
C -3.68168800 3.00993300 -2.24524200
C -2.46509600 2.15697200 -0.33572100
C -3.21217800 3.16326600 -0.92809300
O -4.40267500 4.04243800 -2.73336700
C -3.05679400 -2.17075800 -0.97035200
C -3.49614800 -3.43774600 -1.32278400
C -2.57149700 -4.47126000 -1.55715900
C -0.76971000 -2.93260600 -1.06306300
C -1.20000500 -4.21035700 -1.42531000
O -3.10312500 -5.66705800 -1.90141200
C 3.07882000 -2.17091600 0.83879700
C 4.08399100 -3.09864800 0.61824600
C 5.11188900 -2.81915000 -0.30123700
C 4.09177200 -0.67224300 -0.75810900
C 5.11122900 -1.59513600 -0.98908900
O 6.04693000 -3.78302300 -0.44334300
C 3.56914300 2.25370800 0.71039400
C 4.01659600 3.56234300 0.61293200
C 3.22615300 4.53848300 -0.01973200
C 1.53459800 2.85815400 -0.43506700
C 1.97688800 4.17790800 -0.54433800
O 3.75726700 5.78243700 -0.06807000
C -4.93076600 3.95472200 -4.05520200
C -2.22796400 -6.76144700 -2.15584100
C 7.13301700 -3.56711900 -1.34197700
C 3.01464700 6.82328700 -0.69469200
H 0.68613900 -1.20095000 4.56143500
H -3.82571700 -1.96831100 4.49922100
H -1.73157000 -1.41196100 4.26224100
H -3.09755000 0.74404800 2.67627300
H -1.70996000 1.64249100 4.75903300
H -2.68812200 0.53698700 5.70364900
H 0.51982300 0.62097800 4.84916000
H -5.43187000 -2.06009700 3.78825000
H -4.03648600 -1.74136000 2.75043100
H -5.06282600 0.15354200 4.95903300
H -3.79026100 1.91828000 3.78051100
H -5.47646200 0.31823300 3.26411800
H -2.43270800 -0.07999300 -2.90050500
H -3.74430400 1.68781300 -3.96524100
H -2.11826200 2.29114400 0.68650900
H -3.45743600 4.07587100 -0.39453700
H -3.79208500 -1.38891700 -0.80243500
H -4.55375300 -3.65663600 -1.42941100
H 0.29659300 -2.75067200 -0.95992900
H -0.46501400 -4.98697900 -1.60216000
H 2.29883500 -2.40105100 1.56161900
H 4.10902100 -4.04448700 1.14970000
H 4.11753700 0.27585600 -1.28701900
H 5.89575900 -1.35369800 -1.69663300
H 4.20216200 1.51894900 1.20006100
H 4.97966000 3.85866800 1.01604800
H 0.55916200 2.60299500 -0.83948000
H 1.34631400 4.90918600 -1.03653600
H 0.72445500 0.94421100 -2.66712800
H 1.85899100 -0.40270000 -2.39835000
H 0.17013600 -0.74508900 -2.78879400

H -5.47085800 4.88762300 -4.21879400
H -4.12960600 3.86122600 -4.79827000
H -5.62330200 3.10991700 -4.15097600
H -2.87309900 -7.60257900 -2.41161700
H -1.55648100 -6.54798100 -2.99669800
H -1.63554300 -7.01327100 -1.26748600
H 7.74812600 -4.46528400 -1.28128100
H 6.77758900 -3.43648300 -2.37123000
H 7.72809600 -2.69573900 -1.04356400
H 3.63360100 7.71754400 -0.61486800
H 2.83051500 6.60054200 -1.75303500
H 2.05888800 6.99474400 -0.18400000

2INT4

N 0.18686800 -0.06533000 -0.99884900
P 1.48916600 -0.38561900 0.10988000
P -1.27780600 0.29158600 -0.16202300
Cr -0.43671500 -0.28883700 2.11316500
C 0.34703100 0.08598600 -2.45416700
C 2.39800300 -1.83039000 -0.53996500
C 2.66708200 1.00675600 -0.10145800
C -2.53995600 -0.80790600 -0.88329900
C -1.81503200 1.97972300 -0.59516000
C 2.22791800 -3.08103400 0.08884600
C 2.86675100 -4.21726700 -0.38273900
C 3.70454300 -4.13987600 -1.50957000
C 3.25212300 -1.76663800 -1.65470000
C 3.89761900 -2.90138900 -2.14320800
O 4.27888900 -5.29996000 -1.89343000
C 2.21037800 2.29236200 -0.45573700
C 3.07672000 3.37598900 -0.49351300
C 4.43346300 3.21314300 -0.16230900
C 4.02173900 0.86059400 0.23437800
C 4.90211500 1.94360500 0.20600900
O 5.19366400 4.33040600 -0.23068300
C -2.17714400 -2.06126900 -1.41490500
C -3.14112300 -2.94671600 -1.87090600
C -4.50572500 -2.61483000 -1.78990200
C -3.90373600 -0.48910700 -0.80194900
C -4.88315100 -1.37712800 -1.24786400
O -5.36467900 -3.54788000 -2.25852000
C -1.58898900 3.03067700 0.31672100
C -1.92838600 4.33802100 0.00586700
C -2.50890700 4.63885500 -1.23967100
C -2.40293800 2.29446100 -1.83173700
C -2.74635400 3.60450000 -2.15988500
O -2.80328200 5.93927700 -1.44919700
C 5.14772000 -5.30251600 -3.02408900
C 6.57919500 4.24031900 0.08874400
C -6.76446700 -3.28716900 -2.20121000
C -3.40672300 6.32396500 -2.68305300
H 1.59434600 -3.16270500 0.96668500
H 2.74209400 -5.17927900 0.10368700
H 3.43710300 -0.81438900 -2.14283900
H 4.54827900 -2.81253000 -3.00532000
H 1.16659200 2.44817000 -0.71306400
H 2.72979800 4.36339600 -0.78126500
H 4.41207800 -0.11476200 0.51189600
H 5.94274400 1.78687000 0.46504200
H -1.12927500 -2.33939300 -1.47812700
H -2.86756800 -3.90769000 -2.29457200
H -4.21834900 0.46663900 -0.39281200
H -5.92688000 -1.09555400 -1.17306400
H -1.15060300 2.81750800 1.28710200
H -1.76460200 5.14827000 0.70894600
H -2.61224100 1.50747900 -2.55000100
H -3.19863400 3.80952300 -3.12311100
H -0.60945900 -0.10165600 -2.94905900
H 0.69871800 1.08761300 -2.72620300
H 1.06233600 -0.65469000 -2.82200800
H 5.47998400 -6.33474200 -3.13742500
H 4.61968300 -4.98683700 -3.93194900
H 6.01723600 -4.65501300 -2.85866400
H 6.97737600 5.24768500 -0.03661900
H 6.72903200 3.91531200 1.12570500
H 7.10092300 3.55475300 -0.59028600
H -7.24643200 -4.16911000 -2.62413800
H -7.10142300 -3.14757200 -1.16678000
H -7.03003800 -2.40526600 -2.79694400
H -3.54896400 7.40286200 -2.61689100
H -2.75428100 6.09277400 -3.53362800
H -4.37864500 5.83479200 -2.81907200
C -1.02136200 -2.22948100 1.99213900
C -0.38236300 -2.40299300 3.34894400
C -1.31695900 -2.68659800 4.53774000
C -2.53712300 -1.75700500 4.66918600
C -2.24044900 -0.24668900 4.59361100
C -2.09603800 0.30920500 3.16768400

H -2.10572300 -2.32824100 1.96403800
H -0.55384400 -2.78764900 1.18041400
H 0.43990900 -3.12880400 3.32349900
H 0.18696200 -1.45208900 3.64053400
H -1.66585500 -3.72357600 4.45428100
H -0.72337400 -2.63384800 5.46047100
H -3.01298700 -1.98347200 5.63108100
H -3.28341200 -2.00631900 3.90292600
H -3.06673500 0.28293700 5.09160400
H -1.34813100 -0.01513700 5.19796600
H -2.97942300 0.04111600 2.57157600
H -2.04602700 1.40893200 3.18922900
C 1.46368600 0.84140100 3.79581000
C 0.90507900 1.87199100 3.13865800
H 2.32127500 0.30895900 3.39102300
H 1.12573300 0.54802800 4.78654300
H 1.29834700 2.21431700 2.18432600
H 0.09284600 2.44422400 3.57799600

2TS4

N 0.20987600 0.22726300 -0.94366100
P -1.20539200 0.60601500 -0.01835600
P 1.48759800 -0.28919200 0.10936900
Cr -0.39352900 -0.53333800 2.12351800
C 0.62924500 -2.16829500 3.17688700
C -0.22882300 -2.98226900 2.17789200
C -1.31422400 -3.87883900 2.80271000
C -2.28540800 -0.66461300 2.96382100
C -2.64278100 -1.88510200 3.82256500
C -2.67758200 -3.21505700 3.04921700
C 0.60879000 -0.37674000 4.24536000
C 0.35737000 0.82073300 3.51202900
C 0.43077800 0.72957600 -2.31092400
C -1.52559500 2.39529100 -0.12641200
C -2.58546300 -0.19553700 -0.90644800
C 2.84198000 0.92647300 -0.07876100
C 2.14516500 -1.84284800 -0.60566000
C -1.28211000 3.20189800 1.00357300
C -1.46320800 4.57529600 0.95704300
C -1.89284400 5.19091200 -0.23228500
C -1.96091100 3.02299400 -1.30600300
C -2.14230000 4.40328400 -1.36850600
O -2.03943100 6.53159900 -0.17750500
C -2.40306900 -1.47893500 -1.46121400
C -3.45890300 -2.16613100 -2.03820700
C -4.74657500 -1.59880500 -2.06138200
C -3.87590000 0.35297400 -0.92345900
C -4.94970300 -0.33283100 -1.49440700
O -5.70704300 -2.34963200 -2.64553000
C 2.70084200 2.18153700 0.55170400
C 3.67825000 3.15584200 0.43623900
C 4.84441800 2.90255100 -0.30994900
C 4.00481900 0.69145100 -0.82587800
C 5.00183500 1.66095400 -0.94275600
O 5.74123600 3.91265200 -0.35390600
C 3.22486600 -2.47776600 0.04556800
C 3.70167200 -3.70577000 -0.38673100
C 3.10687900 -4.35043100 -1.48734000
C 1.55772100 -2.50007200 -1.69294000
C 2.02787300 -3.73844800 -2.13919800
O 3.64461500 -5.54378500 -1.82914800
C -2.48470900 7.22923800 -1.33889600
C -7.03989000 -1.84663100 -2.69501400
C 6.94953200 3.73138800 -1.08779600
C 3.09825200 -6.25192100 -2.93806500
H 1.66649400 -2.12135000 2.84274800
H -0.72633400 -2.38819100 1.35970200
H 0.48292700 -3.57849200 1.59464200
H -1.46308200 -4.74123600 2.14128300
H -2.40892800 0.26018500 3.54376900
H -2.98502300 -0.60581900 2.11709100
H -1.96635900 -1.97733800 4.68568100
H -3.64128400 -1.72157100 4.25570500
H -3.30185600 -3.93691800 3.58994400
H -3.17695300 -3.04593700 2.08380900
H -0.92368900 -4.28416700 3.74543100
H 0.58181600 -2.64967700 4.15087500
H 1.63454000 -0.62694500 4.49836500
H -0.11647800 -0.66825800 4.99852100
H 1.21584400 1.35467600 3.10918900
H -0.47855000 1.44154000 3.82447700
H -0.95554200 2.74270600 1.93159900
H -1.28639700 5.19917700 1.82719000
H -2.17709700 2.43056800 -2.19016700
H -2.47900000 4.85372700 -2.29488800
H -1.41994100 -1.94023400 -1.44472700
H -3.31987500 -3.14814100 -2.47910900
H -4.05818600 1.33264200 -0.49284200

H -5.93101900 0.12692600 -1.49302000
H 1.80519400 2.40630200 1.12476600
H 3.57137700 4.12342200 0.91595700
H 4.14669200 -0.26129100 -1.32576600
H 5.88792500 1.44156700 -1.52692200
H 3.71045200 -1.99873600 0.89313400
H 4.53662900 -4.19151500 0.10803000
H 0.72003500 -2.04756800 -2.21355900
H 1.55120300 -4.20967600 -2.99087500
H 1.31876700 0.25612200 -2.73605200
H -0.42950500 0.47781100 -2.93979200
H 0.57836000 1.81539700 -2.32098500
H -2.52905100 8.28005100 -1.05165200
H -1.78036200 7.10724100 -2.17057500
H -3.48190300 6.89233100 -1.64661400
H -7.62615900 -2.61414700 -3.20110800
H -7.44181100 -1.68498800 -1.68742500
H -7.09143800 -0.91183500 -3.26638200
H 7.50522100 4.66282600 -0.97558000
H 7.54109000 2.90197400 -0.68160700
H 6.74652400 3.55309300 -2.15081800
H 3.68610700 -7.16631200 -3.02326900
H 2.04568000 -6.50957300 -2.76680000
H 3.18867700 -5.67224400 -3.86491400

2INT5

N -0.09879900 -0.05940500 -1.08807700
P 1.23723300 -0.69042300 -0.16796000
P -1.31026800 0.62876300 -0.07735700
Cr -0.22945100 -0.12143300 2.06713500
C -0.14143600 -0.04462700 -2.56048000
C 1.31314300 -2.46458300 -0.61489000
C 2.75597000 0.04307500 -0.86147600
C -2.91599200 -0.01070700 -0.64177800
C -1.34514400 2.42365300 -0.37923400
C 2.50247900 -3.19872000 -0.42532200
C 2.53380400 -4.56978400 -0.62694000
C 1.37189700 -5.25754800 -1.02204200
C 0.15818200 -3.16359200 -0.99248400
C 0.17908700 -4.54374100 -1.20496400
O 1.51152600 -6.59161300 -1.19764700
C 3.32787500 1.15374500 -0.20667600
C 4.45392000 1.78282500 -0.71431500
C 5.04984800 1.31573400 -1.89987800
C 3.36303600 -0.41387500 -2.04261200
C 4.49630200 0.20835200 -2.56330700
O 6.14380800 1.99234300 -2.31078300
C -3.38600300 -1.23534700 -0.12174600
C -4.57176800 -1.79382900 -0.56806100
C -5.33468300 -1.13983300 -1.55421800
C -3.68642300 0.63111900 -1.62509800
C -4.88328500 0.08047200 -2.08134100
O -6.47501000 -1.76199800 -1.91757000
C -2.43720800 3.19999400 0.06276800
C -2.42439700 4.57952800 -0.06367000
C -1.31343900 5.23096000 -0.63166700
C -0.23636500 3.08361800 -0.92560000
C -0.21513800 4.47240400 -1.06262600
O -1.40443400 6.57646500 -0.71481600
C 0.37826800 -7.35553700 -1.59856300
C 6.81864000 1.56661400 -3.49282700
C -7.31438600 -1.15527700 -2.89901100
C -0.31691700 7.30916800 -1.27284200
H 3.41609100 -2.69190500 -0.12710800
H 3.44860300 -5.13734900 -0.48980300
H -0.77682400 -2.62921500 -1.13731600
H -0.73030600 -5.04673500 -1.51265500
H 2.88804800 1.52387400 0.71505100
H 4.90305900 2.63224500 -0.20987200
H 2.95830900 -1.27732900 -2.56207300
H 4.94027600 -0.17338000 -3.47524000
H -2.81973200 -1.75261200 0.64747200
H -4.94252300 -2.73059800 -0.16489000
H -3.35873700 1.57997200 -2.03826800
H -5.45324300 0.60491500 -2.83918000
H -3.30846200 2.72143500 0.50154500
H -3.26322100 5.18270400 0.26812700
H 0.62935200 2.51505600 -1.25200400
H 0.65360100 4.94850200 -1.50187400
H 0.46376200 0.76916200 -2.97445300
H 0.23160000 -0.99674700 -2.94859700
H -1.17485600 0.07460200 -2.89688900
H 0.72810400 -8.38550900 -1.67476200
H -0.42527900 -7.29747200 -0.85392300
H 0.00000100 -7.02569200 -2.57405400
H 7.66001000 2.24948400 -3.61335200
H 7.19236800 0.54094700 -3.38915900
H 6.16455900 1.63425900 -4.37054500

H -8.16300400 -1.82939600 -3.01700200
H -7.67046400 -0.17391100 -2.56392700
H -6.79295900 -1.05220300 -3.85815400
H -0.61644800 8.35682400 -1.23390300
H 0.59937900 7.16926800 -0.68639100
H -0.13530600 7.01976100 -2.31513700
C -1.86415100 -0.40829300 3.23201700
C -1.53511500 -0.49468700 4.72876800
C -0.53881000 -1.61415300 5.07231100
C 0.87169500 -1.51410500 4.46034200
C 1.88242700 -0.56761400 5.13002600
C 1.69558200 0.95534600 5.00146600
C 1.82408500 1.53593700 3.57561200
C 0.54327900 1.63709000 2.74153200
H -2.61861500 0.36284000 3.03141400
H -2.27579300 -1.37178000 2.87582600
H -1.16619400 0.47290200 5.08771200
H -2.45981600 -0.68410200 5.29539300
H -0.42359300 -1.68496800 6.16204700
H -0.98158800 -2.56829000 4.75370600
H 1.31526400 -2.51771800 4.45863600
H 0.82563900 -1.28948600 3.36352900
H 1.90510700 -0.82700400 6.19728700
H 2.88041500 -0.81566200 4.74227300
H 0.74559300 1.27454400 5.44648200
H 2.47744800 1.41180100 5.62088100
H 2.22405600 2.55828300 3.66963400
H 2.60049900 0.97843500 3.02740600
H -0.27362400 2.11474600 3.29863800
H 0.71311100 2.21333500 1.82199700

2TS5

N -0.29283900 0.18235400 -1.12774200
P 1.02350900 0.79493800 -0.19530300
P -1.36430100 -0.58198000 -0.01298700
Cr 0.04766200 -0.18259400 1.99606300
C -0.50665600 0.37818100 -2.56731300
C 2.52924400 0.06792300 -0.93126900
C 1.14988300 2.58953600 -0.49068100
C -1.75510500 -2.22917100 -0.68001200
C -2.93127700 0.35299300 -0.03298800
C 3.79173600 0.67246500 -0.75710700
C 4.94567000 0.05608300 -1.21569000
C 4.87702100 -1.19151900 -1.86174300
C 2.47570100 -1.18449000 -1.56012200
C 3.62997900 -1.81108400 -2.03189400
O 6.05796500 -1.70229500 -2.27694100
C 0.53216700 3.47099900 0.42153600
C 0.53976400 4.84125400 0.21365000
C 1.17171300 5.37873600 -0.92273200
C 1.77514700 3.14103800 -1.62091000
C 1.79096200 4.51687500 -1.84279700
O 1.12946300 6.72322400 -1.03420700
C -0.97328400 -3.33070200 -0.27372300
C -1.18212500 -4.59247100 -0.80615700
C -2.18858200 -4.79540600 -1.76902100
C -2.75619100 -2.44608500 -1.64058800
C -2.97751500 -3.71094500 -2.18433100
O -2.31592500 -6.06008100 -2.22292000
C -4.13366600 -0.23548600 0.41212000
C -5.29764600 0.51071800 0.51017200
C -5.29795200 1.87587700 0.16996800
C -2.94026800 1.71789000 -0.35304700
C -4.10757500 2.47773800 -0.26360900
O -6.48547400 2.51001200 0.29368900
C 6.06899200 -2.96262100 -2.94127800
C 1.76653400 7.34738700 -2.14749600
C -3.32535500 -6.35207800 -3.18765400
C -6.56496400 3.89496000 -0.03109300
H 3.87307300 1.63792500 -0.26647700
H 5.91847800 0.52050200 -1.09066900
H 1.51976000 -1.68305100 -1.69432400
H 3.54745400 -2.77155900 -2.52710000
H 0.04021400 3.07795700 1.30815700
H 0.07391600 5.52302200 0.91775400
H 2.26846000 2.49278300 -2.33890200
H 2.28484500 4.90671200 -2.72512600
H -0.19501600 -3.19734500 0.47361900
H -0.58939200 -5.44514800 -0.49111800
H -3.38562100 -1.62259100 -1.96434200
H -3.76119500 -3.84219300 -2.92116000
H -4.16279300 -1.28913600 0.67450900
H -6.22700800 0.06130500 0.84486000
H -2.02698900 2.20644100 -0.68006500
H -4.07895100 3.52714500 -0.53271000
H -1.02388600 1.32073500 -2.77893900
H 0.45697400 0.37808900 -3.08455600
H -1.10225200 -0.44989800 -2.96309400

H 7.11445500 -3.16223500 -3.17812800
H 5.68356300 -3.75846100 -2.29212300
H 5.48459300 -2.92902500 -3.86896100
H 1.60851800 8.41788500 -2.01441600
H 2.84224300 7.13523600 -2.15915100
H 1.31623900 7.02642100 -3.09452400
H -3.23819300 -7.41974300 -3.39041800
H -4.32533800 -6.13513800 -2.79358300
H -3.16332000 -5.78941500 -4.11488300
H -7.60287500 4.17991500 0.14344200
H -5.90899800 4.49329000 0.61311700
H -6.30963100 4.07260000 -1.08300300
C -1.39212600 -1.33156300 2.99008100
C -0.42803500 -1.01876400 4.02267600
C 0.46877300 -2.14338500 4.56375100
C 1.51712100 -2.66298200 3.54503700
C 2.98124400 -2.34489900 3.89748700
C 3.34606000 -0.87698100 4.18931000
C 3.05960500 0.15697800 3.07701200
C 1.69216000 0.85570000 3.16982100
H -2.35392200 -0.81952400 3.01173700
H -1.45014800 -2.35706900 2.62601000
H 0.57990600 -0.12460300 3.60524000
H -0.79425700 -0.32939000 4.79005400
H 0.98049900 -1.78864900 5.46521800
H -0.18112100 -2.96435800 4.88866900
H 1.42515000 -3.74959000 3.43190100
H 1.29312400 -2.26009200 2.54013200
H 3.25594700 -2.93932400 4.77956000
H 3.61680800 -2.71037400 3.07897300
H 2.86007600 -0.54407900 5.11734200
H 4.42088400 -0.86169200 4.40758700
H 3.82850600 0.94040600 3.14313800
H 3.20234600 -0.30741700 2.09134400
H 1.55202800 1.24148900 4.18966800
H 1.65558200 1.73955900 2.52556800

2SC3

N -0.70899400 -0.13171500 -1.08688900
P -1.73344700 0.14504300 0.28395700
P 0.91075100 -0.26445800 -0.49750100
Cr 0.07396400 -0.10131000 1.87526400
C -1.31349900 0.18861500 3.55318500
C -0.10371600 -0.38959400 3.95612400
C 1.08225900 0.38835900 4.49812400
C 4.77834000 0.87987000 3.14937900
C 3.58421100 0.91006100 4.11832400
C 2.33190600 0.17516200 3.61889300
C 5.29181900 -0.51610200 2.75047300
C 5.76736900 -1.37942000 3.92501900
C -1.11733200 -0.14261300 -2.49821400
C -2.40924700 1.83241700 0.11814300
C -3.15280400 -0.98136600 0.12980200
C 1.95824200 0.96162500 -1.34217700
C 1.51078800 -1.89646500 -1.06183000
C -3.57829900 2.21568600 0.80740200
C -4.02744000 3.52641800 0.77153700
C -3.31733500 4.50189000 0.04781700
C -1.70109700 2.81622900 -0.58615400
C -2.14665700 4.13809200 -0.63360700
O -3.84302300 5.74754600 0.07262700
C -3.10757300 -2.22501000 0.79326900
C -4.13806500 -3.14228700 0.66622400
C -5.25688900 -2.84088100 -0.13284400
C -4.27537200 -0.69303500 -0.66314700
C -5.31996400 -1.60640500 -0.79911500
O -6.20950700 -3.79552300 -0.18836100
C 2.27909400 2.16072100 -0.67289900
C 3.03462500 3.14618100 -1.28867900
C 3.49670300 2.96118000 -2.60479500
C 2.42820400 0.79013000 -2.65444600
C 3.18757400 1.77252300 -3.28669800
O 4.22681500 3.97454800 -3.11647700
C 2.89071800 -2.16433000 -1.17318900
C 3.34603900 -3.43904600 -1.47251500
C 2.43439900 -4.49256200 -1.66746900
C 0.61210100 -2.95752400 -1.24328200
C 1.05890100 -4.24312400 -1.55226200
O 2.98107000 -5.69413900 -1.95981800
C -3.18150100 6.78991900 -0.63804500
C -7.38463300 -3.55748000 -0.96095300
C 4.74999600 3.85433100 -4.43830100
C 2.12099700 -6.81095700 -2.16703300
H -1.48267500 1.25919900 3.67202100
H 4.94791100 -1.63733200 4.60640100
H -0.08002400 -1.47065700 4.12611000
H 0.84108000 1.45934200 4.54185700
H 5.60321600 1.44329700 3.60588200

H 4.50574500 1.42990400 2.23617900
H 3.86801400 0.47540900 5.08567700
H 3.32237700 1.95730700 4.32145000
H 2.14008100 0.53670900 2.57959000
H 2.53681400 -0.90070900 3.53123700
H 1.30710300 0.08156600 5.52949900
H -2.21505200 -0.41206800 3.47095400
H 6.20263600 -2.31965400 3.56905400
H 6.53473600 -0.86054800 4.51214000
H 4.51509900 -1.05625400 2.18996100
H 6.12350800 -0.38066200 2.04698400
H -4.14826500 1.48037200 1.36825800
H -4.93032100 3.82597200 1.29382300
H -0.78689900 2.55667800 -1.11213500
H -1.58086900 4.86880500 -1.19963900
H -2.25550700 -2.47264900 1.42226200
H -4.11329500 -4.09685400 1.18178900
H -4.34812400 0.26291000 -1.17312200
H -6.17275700 -1.34933500 -1.41635900
H 1.93466900 2.32205600 0.34615800
H 3.29155100 4.06686500 -0.77497700
H 2.21413200 -0.12904900 -3.19191900
H 3.53546900 1.60521000 -4.29929800
H 3.61585200 -1.36753500 -1.03415200
H 4.40652100 -3.64945800 -1.56637800
H -0.45674300 -2.78361200 -1.15406400
H 0.33409600 -5.03495300 -1.70129300
H -0.44560000 -0.78915200 -3.06999500
H -2.12901500 -0.55111100 -2.58238200
H -1.10348300 0.86212400 -2.93569900
H -3.78064900 7.68594500 -0.47321500
H -2.16737300 6.95352000 -0.25310000
H -3.13685600 6.57367400 -1.71258500
H -7.99791100 -4.45157000 -0.84644500
H -7.93526700 -2.68601800 -0.58718800
H -7.14120500 -3.41431700 -2.02058400
H 5.30065500 4.77726400 -4.62154200
H 5.43195600 2.99948700 -4.51877900
H 3.94527600 3.75596500 -5.17676800
H 2.77839000 -7.65422500 -2.38080000
H 1.52805300 -7.02879300 -1.27021400
H 1.45120000 -6.64510100 -3.01974000

2P′

N 1.01138300 0.12448500 -0.81367300
P 1.82146200 -0.61937000 0.52756100
P -0.55157700 0.73386100 -0.38049900
Cr -0.14460600 0.06611600 2.11358800
C -0.17018800 -0.12097900 4.42720900
C -1.44213700 0.31902200 4.18510400
C -2.66901100 -0.55730300 4.16101100
C -5.93679200 -0.87129200 1.96856800
C -4.93756700 -1.10643900 3.11540000
C -3.68050900 -0.22750000 3.04905600
C -6.58702000 0.52332100 1.91712900
C -7.44815300 0.86559100 3.13893300
C 1.53175100 0.13343200 -2.19111900
C 1.99575200 -2.39125100 0.10531300
C 3.51861100 0.04462900 0.54898500
C -1.76990500 -0.01620000 -1.51349100
C -0.51228900 2.50961500 -0.81912000
C 2.97216700 -3.19158800 0.73348500
C 3.02115100 -4.55979200 0.51481400
C 2.08833100 -5.17727300 -0.33760800
C 1.06065500 -3.02114600 -0.72721900
C 1.10307400 -4.39729900 -0.95948300
O 2.22684700 -6.51530000 -0.48733700
C 3.79174200 1.14694500 1.38505900
C 5.04545200 1.73613600 1.41109100
C 6.07744200 1.23359400 0.59699400
C 4.55954400 -0.44871600 -0.25432100
C 5.82695300 0.13257200 -0.23747600
O 7.26341400 1.87189300 0.69607500
C -2.48812600 -1.14715700 -1.07358700
C -3.38857800 -1.79569700 -1.90446100
C -3.60392100 -1.32592600 -3.21309700
C -1.99638700 0.44075700 -2.82143700
C -2.90070100 -0.19884600 -3.66807300
O -4.50294700 -2.02105700 -3.94298800
C -1.70713400 3.22710400 -1.03291200
C -1.68850400 4.59875600 -1.23358200
C -0.47055100 5.30225900 -1.22262900
C 0.69347000 3.22396100 -0.79383800
C 0.72517400 4.60436700 -1.00100700
O -0.56154400 6.63580900 -1.43385600
C 1.31752000 -7.21013600 -1.33446200
C 8.36545600 1.40943900 -0.08189900
C -4.79152900 -1.59690600 -5.27363600

C 0.63074100 7.41475900 -1.43443400
H 0.03596400 -1.17463800 4.61323600
H -6.85644100 0.91896300 4.06035400
H -1.62216300 1.39644900 4.15926600
H -2.37201800 -1.61347500 4.10786300
H -6.73164600 -1.62570800 2.04551500
H -5.42828000 -1.06147900 1.01199300
H -5.43339000 -0.95273400 4.08328900
H -4.63078800 -2.16200200 3.09999600
H -3.20046900 -0.35439900 2.06569100
H -3.95505000 0.83348700 3.11845700
H -3.17108800 -0.43249400 5.13359600
H 0.63429800 0.57795500 4.65282300
H -7.94065500 1.83583200 3.01163200
H -8.23147900 0.11329300 3.29263400
H -5.81474400 1.29399200 1.78404900
H -7.21221600 0.57228200 1.01606600
H 3.70742400 -2.74040800 1.39398900
H 3.77563100 -5.17841300 0.99008900
H 0.28323200 -2.43679100 -1.21127900
H 0.37178400 -4.84610200 -1.62163800
H 3.00911000 1.54322700 2.02857300
H 5.26206700 2.57972900 2.05848100
H 4.38935700 -1.30981900 -0.89368600
H 6.60723000 -0.27661100 -0.86842100
H -2.34002800 -1.52255800 -0.06353000
H -3.94714100 -2.66305400 -1.56768500
H -1.47124900 1.31786200 -3.18762300
H -3.05198300 0.18427300 -4.67043100
H -2.66099300 2.70736800 -1.05187800
H -2.60475700 5.15431400 -1.40574600
H 1.62928800 2.70085500 -0.61673500
H 1.67802800 5.12067900 -0.98924800
H 1.19596600 1.03841400 -2.70507100
H 2.62503100 0.14774100 -2.16997700
H 1.19906200 -0.74255900 -2.75884700
H 1.62040600 -8.25709200 -1.29802600
H 0.28555400 -7.11637200 -0.97394300
H 1.37940700 -6.84852500 -2.36839900
H 9.19874100 2.06651700 0.16836800
H 8.62567900 0.37534000 0.17384100
H 8.15241800 1.48186600 -1.15524300
H -5.53749300 -2.29716900 -5.65041000
H -5.20465300 -0.58120300 -5.28590700
H -3.89762500 -1.63931300 -5.90761000
H 0.31325600 8.44118800 -1.62091400
H 1.14215400 7.36140100 -0.46533000
H 1.31580900 7.09470500 -2.22928100

2INT6

N 0.47491700 -0.05744800 -1.02738200
P -0.95436300 0.64176200 -0.36163100
P 1.53543500 -0.67656500 0.19887000
Cr -0.48065000 -0.06534000 2.09842100
C -1.60305000 1.43874800 2.92666900
C -3.07508800 1.72795400 2.60690000
C -4.07407300 1.12423800 3.61316400
C -3.86690700 -0.36934700 3.90179500
C -3.93289400 -1.26244400 2.65608100
C -3.36059800 -2.67117500 2.86390700
C -1.85955200 -2.71286600 3.19879600
C -0.92833500 -2.09380500 2.15242600
C 0.59198500 -0.56153400 4.55056700
C 1.37011700 0.33655700 3.92079200
C 0.84853600 0.04024900 -2.44868100
C -0.81654100 2.45226700 -0.58977600
C -2.33384300 0.12408400 -1.43437600
C 3.15409400 0.15011700 -0.02807700
C 1.84289400 -2.41571300 -0.28098800
C 0.44116300 3.06275700 -0.69059500
C 0.57433200 4.45062500 -0.76249800
C -0.56853400 5.26196600 -0.72022100
C -1.95579800 3.28325400 -0.53764000
C -1.83614500 4.66233900 -0.60440200
O -0.56284700 6.61237400 -0.78544400
C -2.69837100 0.83109400 -2.59245100
C -3.71199400 0.37369000 -3.43321200
C -4.38846400 -0.81735800 -3.12592100
C -3.02494200 -1.07026300 -1.14156000
C -4.03359400 -1.53581300 -1.96916400
O -5.38383400 -1.35086900 -3.86463200
C 3.45336800 1.27801300 0.76475400
C 4.64568200 1.96998400 0.61660700
C 5.59093100 1.54888500 -0.33611300
C 4.10878500 -0.25564200 -0.97480300
C 5.31383400 0.42719100 -1.13391000
O 6.72289600 2.28258700 -0.40109100
C 2.95581300 -3.10919500 0.23868100

C 3.13240200 -4.46220800 -0.00601400
C 2.19626300 -5.17208800 -0.77941100
C 0.90879600 -3.13840300 -1.03517400
C 1.07987500 -4.49964200 -1.29591400
O 2.46432100 -6.48577500 -0.96334400
C 0.68387400 7.29129500 -0.90437500
C -5.80912700 -0.67420500 -5.04622800
C 7.74073900 1.90536700 -1.32634500
C 1.55918700 -7.27169200 -1.73218600
H -1.43024800 1.45164600 4.01241200
H -3.23661600 2.81603400 2.58790300
H -3.32243000 1.38415700 1.59339900
H -3.99769900 1.67702500 4.55952700
H -5.09342700 1.29497000 3.24066200
H -2.89287000 -0.49269200 4.39212800
H -1.57484900 -3.77024700 3.32068300
H -1.68381700 -2.26149000 4.18486300
H -3.53141000 -3.26528300 1.95459000
H -3.91251400 -3.17981400 3.66614500
H -4.61270000 -0.70739700 4.63357400
H -0.95338400 2.22594200 2.48970100
H 0.83183400 -1.62089700 4.54624300
H -0.27650600 -0.26052400 5.12894800
H -3.38314100 -0.78582000 1.83439900
H -4.97449200 -1.33584000 2.31505000
H -1.24022800 -2.34826900 1.13031700
H 0.09870400 -2.46818300 2.27578100
H 2.27689900 0.03712000 3.40284600
H 1.16788600 1.40399900 3.98249700
H 1.34016100 2.45526700 -0.72062800
H 1.56363900 4.88334100 -0.85567400
H -2.94727700 2.84879300 -0.45404800
H -2.71062700 5.30427100 -0.57335000
H -2.19423600 1.75859100 -2.84498400
H -3.96636400 0.94740600 -4.31659800
H -2.77480500 -1.63702200 -0.25092700
H -4.57248000 -2.45007200 -1.74263300
H 2.73876700 1.62267000 1.50827200
H 4.87964000 2.83381600 1.23058300
H 3.92084400 -1.12755700 -1.59385600
H 6.02641800 0.08075000 -1.87329000
H 3.69993100 -2.58392000 0.83142000
H 3.99091900 -4.99770100 0.38641500
H 0.03236800 -2.63658700 -1.43586000
H 0.34365200 -5.02127300 -1.89625000
H 1.40805200 -0.85238200 -2.74122200
H -0.05435500 0.08849300 -3.06333600
H 1.46222300 0.92568100 -2.64870700
H 0.43962700 8.35341800 -0.93887300
H 1.32914300 7.09306200 -0.03969300
H 1.20612700 7.00678400 -1.82611000
H -6.61277200 -1.28330800 -5.46068200
H -6.19132900 0.32701200 -4.81420700
H -4.99397300 -0.59872700 -5.77593100
H 8.54525500 2.62842500 -1.18956700
H 8.11570700 0.89692000 -1.11456700
H 7.37720200 1.95447600 -2.35990000
H 1.97495500 -8.27979100 -1.73861700
H 0.56158600 -7.29164100 -1.27614500
H 1.48489900 -6.90152900 -2.76224300

2TS6

N 0.51911000 0.05694600 -1.15478800
P 0.32345200 1.43870300 -0.13809200
P 0.18198200 -1.40917900 -0.29288200
Cr -0.31777100 0.06138100 1.97748800
C -1.28113100 1.53107000 3.03312700
C -2.71276300 1.43323800 2.48529100
C -3.86323400 1.45794400 3.52219500
C -3.83758600 0.36302100 4.60405200
C -3.91668600 -1.09177500 4.07485400
C -2.93998400 -2.08095000 4.74346500
C -1.44540300 -1.69705100 4.64205000
C -0.90576200 -1.61540100 3.21470800
C 1.12611100 -0.74292000 3.70886200
C 1.60686700 0.27490200 2.87935400
C 0.92732500 0.10332300 -2.56845000
C 1.95893600 2.24340800 0.00343000
C -0.70646300 2.61963800 -1.07486900
C 1.57746000 -2.54647200 -0.60319700
C -1.23088600 -2.17529600 -1.17424500
C 3.14107500 1.55352300 -0.29262800
C 4.39466200 2.12916000 -0.07104500
C 4.48221000 3.41796500 0.47339500
C 2.06359500 3.53435200 0.56292900
C 3.30158700 4.11457100 0.79122000
O 5.63672100 4.07405200 0.73076100
C -0.17079000 3.63885400 -1.87740400

C -0.99257400 4.48697900 -2.62016000
C -2.38527400 4.32336900 -2.57591200
C -2.10912200 2.46859700 -1.04532200
C -2.93662300 3.30038600 -1.78163400
O -3.27424600 5.08524400 -3.24834000
C 2.56561300 -2.70134900 0.39013600
C 3.66895800 -3.51600500 0.18839300
C 3.81971100 -4.21013900 -1.02560500
C 1.73896900 -3.25190000 -1.80696300
C 2.84303400 -4.07391300 -2.02574200
O 4.92506000 -4.97925300 -1.12699500
C -1.49506900 -3.55366900 -1.03628200
C -2.63138500 -4.12196400 -1.59148400
C -3.54713600 -3.32806600 -2.30510600
C -2.15905100 -1.39253800 -1.87386500
C -3.30319800 -1.95450200 -2.44436800
O -4.61803900 -3.98055100 -2.81416200
C 6.87388900 3.43080800 0.43971300
C -2.79308700 6.15312200 -4.06227000
C 5.13981300 -5.72919900 -2.32087200
C -5.58482500 -3.24209600 -3.55433300
H -1.20774000 1.29212200 4.09954400
H -2.88155700 2.24824900 1.77277000
H -2.84369900 0.51204600 1.87398700
H -3.85093700 2.43747600 4.01757700
H -4.81359900 1.40089500 2.97288200
H -2.93823300 0.49661100 5.21607600
H -0.85580800 -2.42533700 5.21191800
H -1.30891600 -0.73700500 5.15362000
H -3.09274600 -3.08003700 4.31416100
H -3.18667600 -2.16426600 5.81051500
H -4.67483200 0.54621600 5.28808600
H -0.83370600 2.51657400 2.86818100
H 1.59063600 1.30534100 3.22526900
H 2.32553700 0.05026600 2.09491300
H -3.75639500 -1.10680000 2.98845700
H -4.93424000 -1.47707100 4.21022200
H -1.73983300 -1.36061700 2.51976400
H -0.50298600 -2.56569100 2.85566200
H 0.74365900 -0.49314300 4.69126600
H 1.49750000 -1.75706700 3.60171400
H 3.09582700 0.54821100 -0.70088800
H 5.28714900 1.56900400 -0.32470000
H 1.16801000 4.09705900 0.81372900
H 3.38685000 5.11018800 1.21459800
H 0.90350500 3.78569300 -1.92377400
H -0.54191800 5.26496600 -3.22517600
H -2.55797500 1.69001300 -0.43577600
H -4.01605700 3.19122100 -1.75585100
H 2.46369200 -2.18234400 1.33899600
H 4.42711000 -3.64336400 0.95440400
H 0.98620400 -3.17454300 -2.58571500
H 2.93235700 -4.60396900 -2.96682700
H -0.79932500 -4.19299700 -0.49977800
H -2.83416100 -5.18364900 -1.49375900
H -1.99091400 -0.32569000 -1.99090900
H -3.98894900 -1.31914000 -2.99282200
H 0.28757800 -0.55471500 -3.16436500
H 0.81283900 1.12130100 -2.94942900
H 1.96997500 -0.20728900 -2.69730200
H 7.65045700 4.14066100 0.72617200
H 6.99003000 2.50844700 1.02220500
H 6.96348900 3.20403300 -0.62981800
H -3.68062300 6.62117100 -4.48876500
H -2.24215000 6.88959900 -3.46520800
H -2.15274300 5.77922700 -4.87026000
H 6.07564300 -6.26685000 -2.16627800
H 4.32903600 -6.44792800 -2.48992400
H 5.23655200 -5.07035300 -3.19227400
H -6.33861900 -3.96737200 -3.86240000
H -6.05531700 -2.46807900 -2.93530800
H -5.13842200 -2.77999000 -4.44357100

2INT7

N -0.48698100 -0.00850900 -1.20574700
P -1.28065200 -0.92180800 0.01968200
P 0.81498300 0.95614900 -0.56902300
Cr 0.27265200 -0.22431800 1.85009000
C 0.55343600 -1.89159200 2.97223800
C 2.00692800 -2.17929400 2.55390200
C 3.02302000 -2.43079200 3.69659000
C 3.21331600 -1.26775800 4.68684900
C 3.91627300 -0.02720300 4.09762300
C 3.64189800 1.29795300 4.84204500
C 2.15034700 1.63275400 5.05682800
C 1.27525900 1.53962700 3.79483300
C -0.24083300 1.42524600 4.07324800
C -1.02998700 0.99867100 2.83082400

C -0.90052100 0.00766800 -2.61944100
C -2.98667700 -0.30477400 0.16292300
C -1.41462100 -2.63468700 -0.57904500
C 0.53694700 2.67262000 -1.12143800
C 2.29267600 0.40234400 -1.49931100
C -3.32375500 0.98587300 -0.26552700
C -4.60480600 1.50325800 -0.06866000
C -5.57401400 0.73008300 0.58710300
C -3.96621600 -1.06506400 0.83606800
C -5.23928400 -0.55899000 1.04229600
O -6.84353800 1.12401800 0.83056500
C -2.50776600 -3.09662100 -1.32985300
C -2.54915200 -4.40011600 -1.82346800
C -1.48098900 -5.27512900 -1.57064000
C -0.35018100 -3.52791200 -0.33511400
C -0.37792500 -4.82424500 -0.82110300
O -1.41502300 -6.55480700 -1.99274700
C 0.05079300 3.61510600 -0.19227500
C -0.21881200 4.92178200 -0.56938400
C -0.00902100 5.32985800 -1.89887200
C 0.74873800 3.09699100 -2.44377000
C 0.47991500 4.40635400 -2.83777200
O -0.30026400 6.62073200 -2.16809200
C 3.40985400 1.24999100 -1.64596300
C 4.58065800 0.79172900 -2.23039000
C 4.67539200 -0.53470600 -2.68796200
C 2.40545500 -0.92239600 -1.94338200
C 3.57621500 -1.39295400 -2.54109200
O 5.85732400 -0.87856300 -3.24990500
C -7.25862100 2.41887100 0.40448500
C -2.50211000 -7.09556600 -2.74193600
C -0.09370600 7.11707100 -3.48899600
C 6.02649000 -2.20488800 -3.74013600
H 0.44792100 -1.61238600 4.02685700
H 2.03214500 -3.04178300 1.87661700
H 2.42134700 -1.34854300 1.92906200
H 2.68839800 -3.31701500 4.25039500
H 3.99100500 -2.69044000 3.24607700
H 2.23220800 -0.99520200 5.09311700
H 2.06234100 2.64372100 5.47488500
H 1.73235600 0.96038900 5.81611600
H 4.12624700 2.11201300 4.28628200
H 4.12588900 1.27352800 5.82693500
H 3.78924800 -1.63009500 5.54758900
H -0.13436500 -2.71275600 2.75279800
H -1.96991800 0.48906900 3.06264100
H -1.23700500 1.83053400 2.14361600
H 3.63614000 0.09636100 3.04265400
H 4.99957900 -0.20085400 4.08099800
H 1.62864000 0.63303700 3.25593000
H 1.48181700 2.37724400 3.11504000
H -0.38981400 0.68169700 4.86584400
H -0.60318100 2.38223000 4.47676800
H -2.58233800 1.60597000 -0.76068800
H -4.83450300 2.50046000 -0.42528100
H -3.73445700 -2.06398800 1.19524700
H -5.99993600 -1.14052600 1.55299000
H -3.34779600 -2.43798000 -1.52719500
H -3.41018300 -4.72328300 -2.39659300
H 0.50787100 -3.20433200 0.24678900
H 0.43390700 -5.51821500 -0.62876000
H -0.10860900 3.32190800 0.84156200
H -0.58516200 5.65257000 0.14434000
H 1.14451300 2.40300400 -3.17925900
H 0.65717200 4.69904500 -3.86606200
H 3.36161900 2.28153700 -1.30888300
H 5.44044300 1.44279700 -2.35153600
H 1.56590300 -1.60401300 -1.83571400
H 3.61990300 -2.41900700 -2.88753200
H -0.01568600 -0.01247200 -3.26231100
H -1.49635500 -0.88239000 -2.83851000
H -1.49235200 0.89833800 -2.85685200
H -8.30263700 2.50795600 0.70611300
H -6.66949500 3.20582500 0.89104900
H -7.18319700 2.52249100 -0.68493800
H -2.22708500 -8.12885700 -2.95465000
H -3.43188900 -7.07650000 -2.16115900
H -2.64318900 -6.55332600 -3.68456900
H -0.38594500 8.16701500 -3.45770300
H 0.96005700 7.03971900 -3.78223100
H -0.71925500 6.58538100 -4.21621500
H 7.03987600 -2.24488100 -4.14084000
H 5.92314100 -2.94376800 -2.93577000
H 5.30925900 -2.42916500 -4.53940000
